# Supplementary material for: Pharmacological inactivation of the prion protein by targeting a folding intermediate
Source: Commun Biol. 2021 Jan 12;4:62. doi: 10.1038/s42003-020-01585-x (PMC7804251; doi:10.1038/s42003-020-01585-x)
Supplement: Supplementary file 2 — Supplementary Information [file 42003_2020_1585_MOESM2_ESM.pdf]

Supplementary Figures

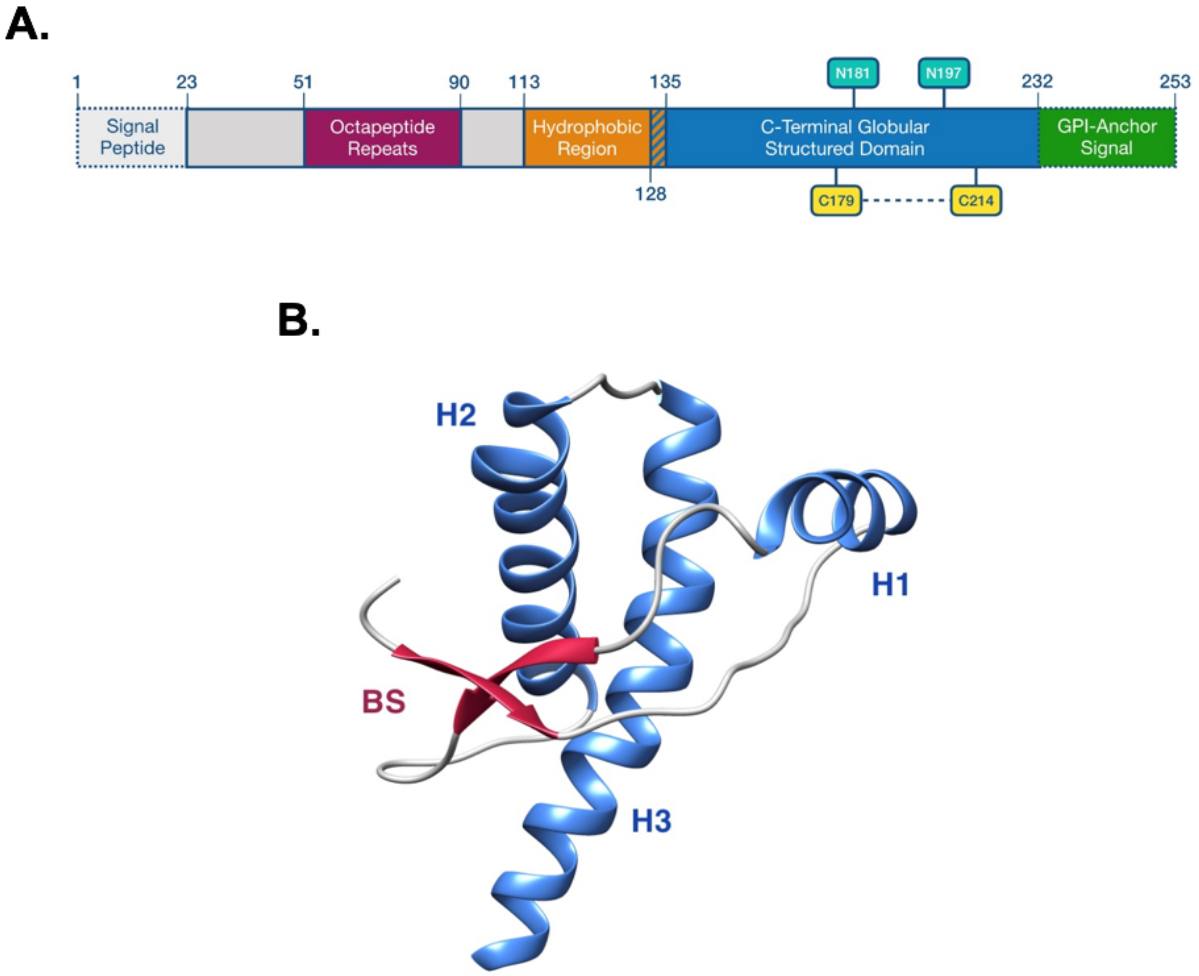

Figure S1: Representation of PrP structure. **A**. The picture illustrates a schematic representation of the structure of human PrP. The protein is organized as follows: a signal peptide (residues 1-22, light gray), removed during the biosynthesis of the protein in the ER, precedes five histidine-containing octapeptide repeats (residues 51-89, purple), which can bind divalent ions. The central region of the molecule includes a highly conserved hydrophobic domain (residues 113-134, orange). The C-terminal, globular domain (solved by NMR, PDB 1QLX, shown in panel (**B**) includes two short anti-parallel β-strands (residues 127–129; and 166–168, light purple) and three α-helices (H1, H2, and H3, residues 143-152, 171-191 and 199-221, respectively; light blue). A C-terminal peptide (residues 232–253, green) is removed to allow the attachment of a glycosylphosphatidylinositol (GPI) moiety, which anchors the protein to the outer leaflet of the plasma membrane. The globular domain also contains two N-linked oligosaccharide chains (at Asn-181 and Asn-197, light green boxes) and a disulfide bond between residues 179 and 214 (yellow boxes).

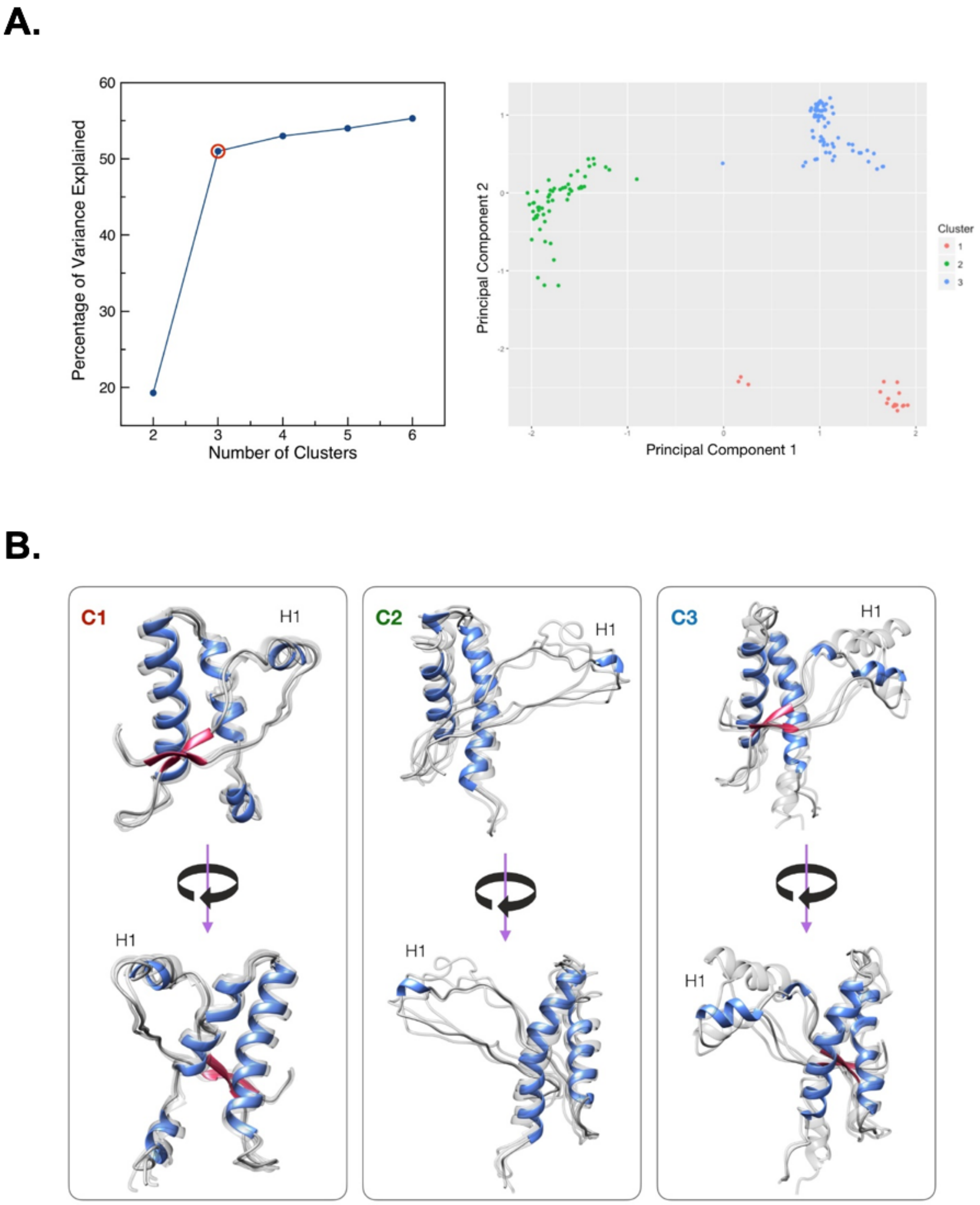

Figure S2: Clustering of PrP folding intermediate conformations. **A**. The left panel shows an elbow plot displaying the percentage of variance explained as a function of the number of clusters. The elbow criterion selects the number of clusters for k-mean at which the marginal gain obtained from the addition of a cluster drops (in this case $k = 3$, red circle). The right panel shows the clustering result represented in the principal component space. Conformations belonging to cluster 1, 2 and 3 are depicted in red, green, and blue, respectively. **B**. Structures corresponding to the three cluster (C) centers (helices, H, colored blue). Configurations obtained from C1, sampled from 2 out of 9 least biased trajectories, are characterized by docking of H1 onto H2 and H3, and an unproperly folded H2-H3 contact region. Both configurations C2 and C3 are characterized by a missing contact region between H1 and H2-H3, as compared to the native state, although the C3 conformations were largely the most represented, being sampled from 7 out of 9 least biased trajectories (while the C2 conformation only in 1 out of 9). Thus, the C3 center was selected for the following in-silico analyses.

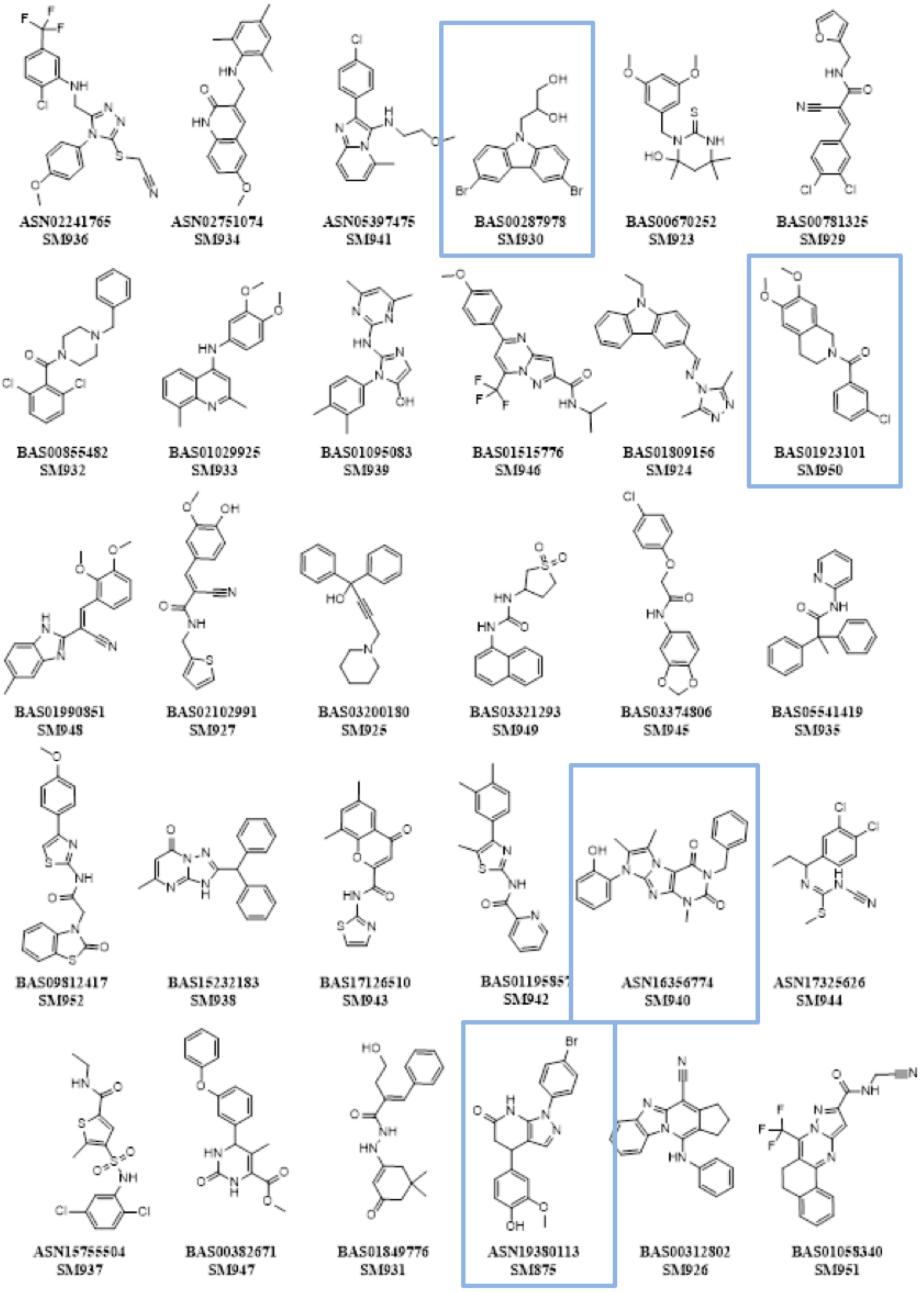

Figure S3: Chemical structures of the 30 virtual hits. The figure illustrates the 2D structure, the Asinex ID code, and the in-house code (SM) for the 30 virtual hits selected for biological evaluation. Each molecule was tested for its ability to lower the level of full-length PrP (assayed by western blotting) without decreasing the expression of NEGR-1, a GPI-anchored protein following the same expression pathway of PrP and used here as control of effect specificity. Four compounds resulted as positive in these analyses: SM875, SM930, SM940, and SM950 (blue boxes).

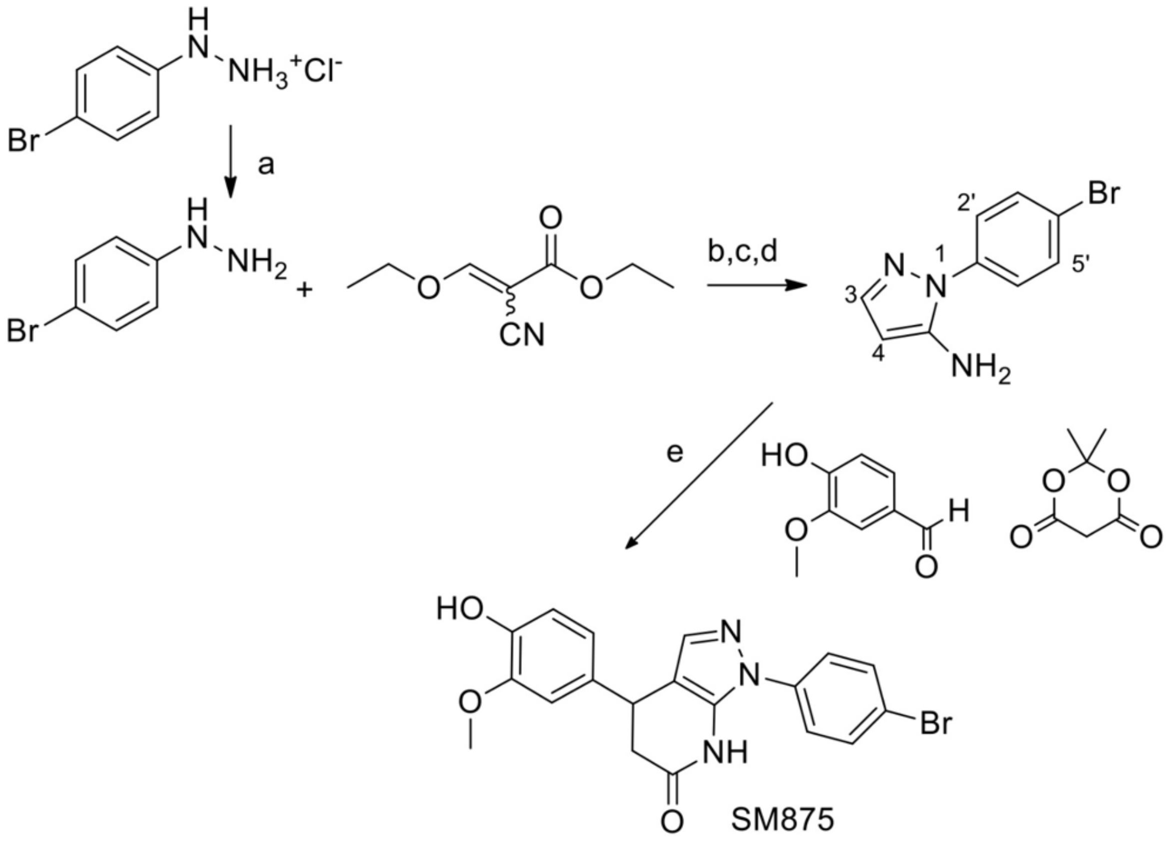

Figure S4: Synthetic scheme of SM875. Reagents and conditions: (a) $NaHCO_3$ aqueous solution; (b) ethanol reflux, 2 h; (c) 1:1 methanol/aqueous 2M NaOH, reflux 1h; (d) 180 °C, 10 min; 61%; (e) ethanol reflux 2.5 h, HPLC purification, 25%. Arbitrary numbering of synthetic intermediate [1-(4-bromophenyl)-1H-pyrazol-5-amine] is used for ^{1}H-NMR assignment: [δH 7.58(d, J 8.7 Hz, H-3'and H-5'), 7.47(d, J 8.7 Hz, H-2'and H-6'), 7.41(s, H-3), 5.62 (s, H-4)].

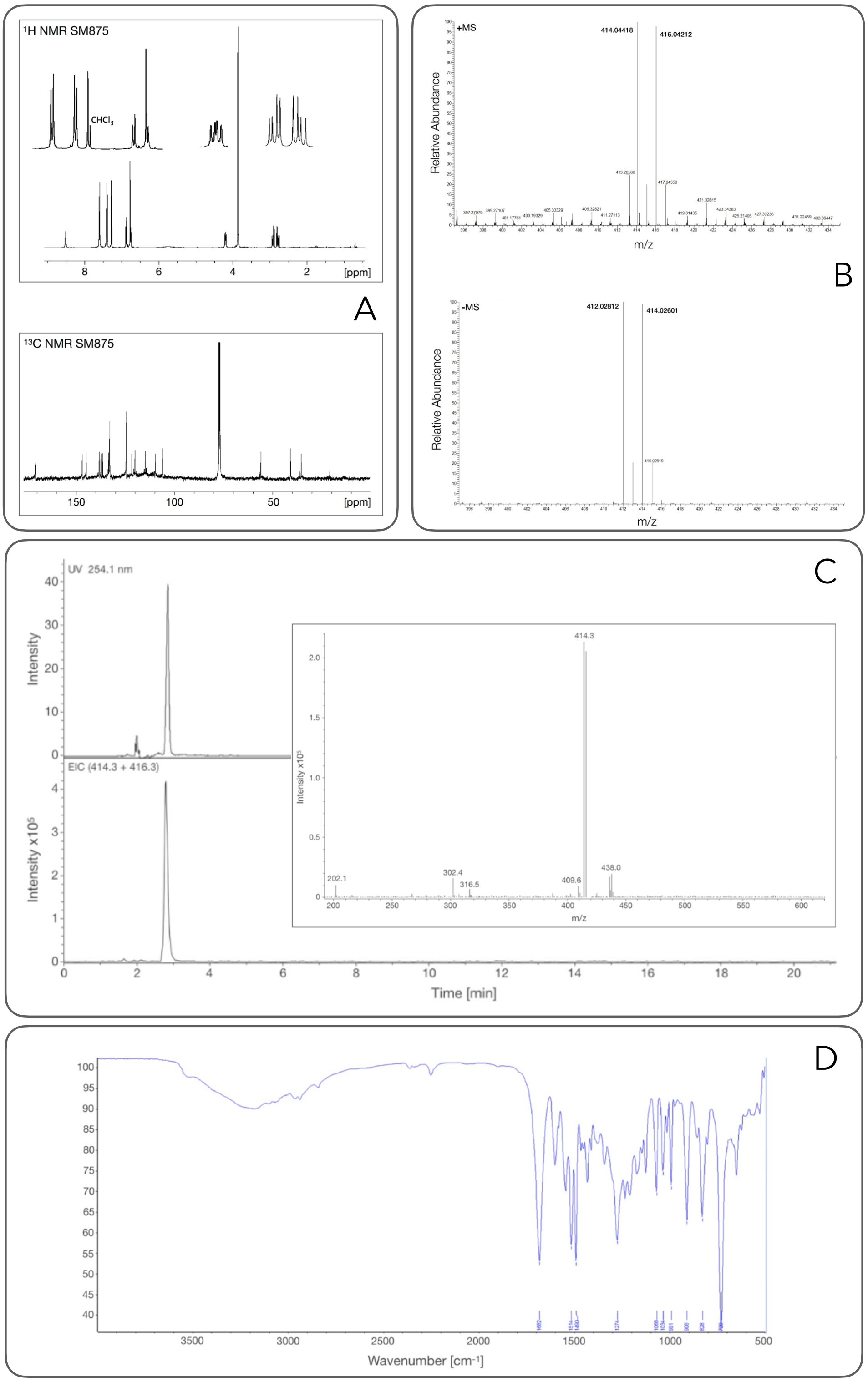

Figure S5: Structural characterization of SM875. **A**. ^{1}H-NMR spectrum of SM875 (upper panel, 400 MHz) in $CDCl_3$ with residual $CHCl_3$ at 7.25 ppm. ^{13}C-NMR spectrum (lower panel, 100 MHz) of SM875 in $CDCl_3$. **B**. Upper panel: high resolution ESI-MS positive ion mode measurement of SM875 (methanol solution) by direct infusion: $[M+H]^+$ ions at m/z 414.0443 (calculated for $C_{19}H_{17}{}^{79}BrN_3O_3$ = 414.0448) and m/z 416.0421 (calculated for $C_{19}H_{17}{}^{81}BrN_3O_3$ = 416.0427). Lower panel: high resolution ESI-MS negative ion mode measurement of SM875 (methanol solution) by direct infusion : $[M\text{-}H]^-$ ions at m/z 412.0282 (calculated for $C_{19}H_{15}{}^{79}BrN_3O_3$ = 412.0302) and m/z 414.0260 (calculated for $C_{19}H_{15}{}^{81}BrN_3O_3$ = 414.0282). **C**. Upper panel: UV chromatogram at l =254 nm of SM875 (LC-ESI(+)-MS-UV; RP18, acetonitrile/water 70:30, flow 1 mL · min^{-1}). Lower panel: MS chromatogram by extracted-ion current of the $[M+H]^+$ peaks at m/z 414 and 416 (1:1) corresponding to the two expected major isotopomers (1:1) $C_{19}H_{17}{}^{79}BrN_3O_3$ and $C_{19}H_{17}$ $^{81}BrN_3O_3$. **D**. FT-IR spectrum of SM875 (cm^{-1}): 1681s, 1543s, 1515s, 1490s, 1274s, 991m, 828m, 728vs.

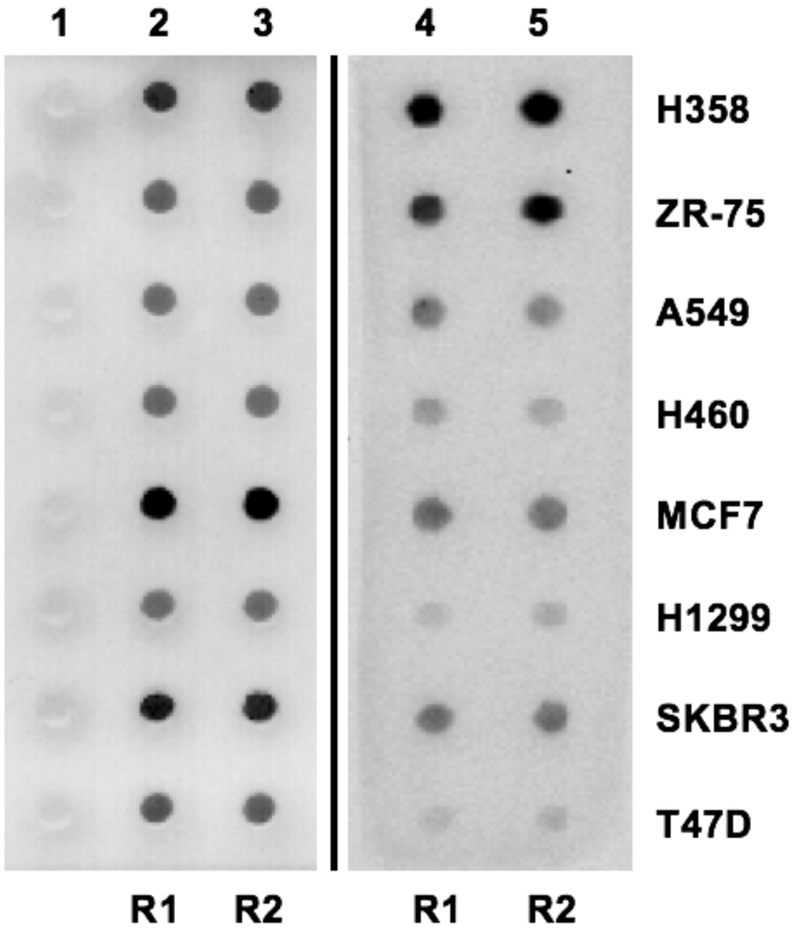

Figure S6: Evaluation of PrP loads in different cancer cell lines. The endogenous expression of PrP in eight different cultured cell lines (indicated) from lung and breast cancers belonging to the Human Tumor Cell Line collection of the National Cancer Institute (NCI) was evaluated by dot-blotting. Cell lysates (lanes 2-5) from two different experiments (R1 and R2) were spotted on to a PVDF membrane, together with a buffer control (lane 1), stained with Ponceau red (lanes 1-3) or incubated with anti-PrP primary antibody (D18) followed by an HRP-conjugated secondary antibody (lanes 4-5). ZR-75 show the highest expression of PrP among the different cell lines.

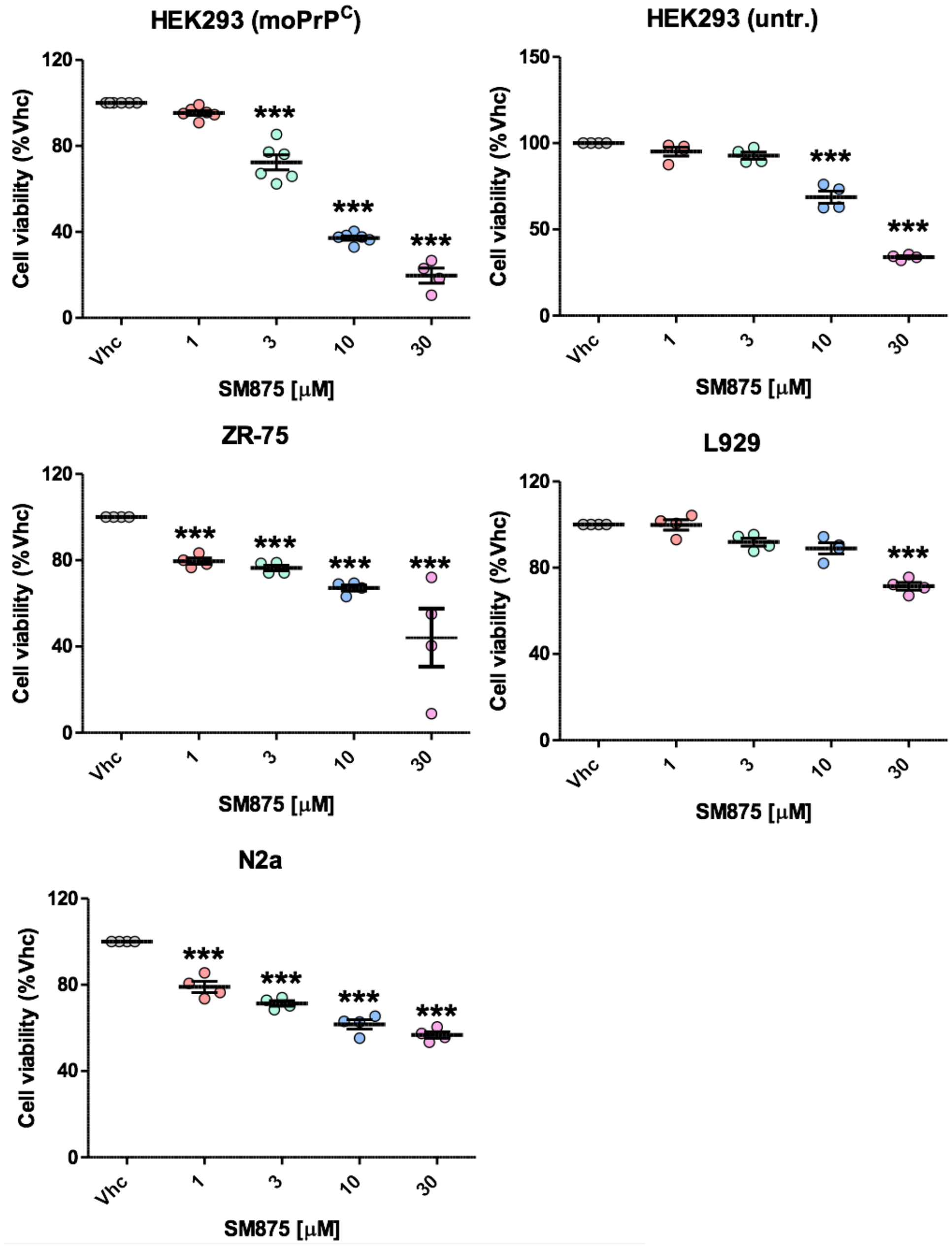

Figure S7: Estimation of SM875 cytotoxicity. In several experiments, we noticed evident cytotoxicity of SM875 at concentrations near to those at which the compound exerted PrP-lowering effects. While experimental controls in the different assays assess effect specificity, the observed cytotoxicity may still represent an important confounding factor. Therefore, we quantified the toxicity of the molecule in the different cell lines, and at the same incubation time and concentrations employed in the study, by using the MTT cell viability assay. Results show that SM875 variably affects cell viability in the different cell lines. In particular, the compound shows high cytotoxicity in moPrP-transfected HEK293 (∼28% reduction of cell viability at 3 μM), N2a (∼21% cell death at 1 μM) and ZR-75 cells (∼24-33% reduction between 1 and 10 μM). Conversely, SM875 appears to be much less toxic in untrasfected N2a cells (no significant alteration of cell viability at 3 μM, ∼32% reduction at 10 μM) and L929 fibroblasts (cell viability almost completely unaltered up to 10 μM, ∼29% reduction at 30 μM). These results clearly indicate that SM875 needs to be optimized to increase its potency and reduce its intrinsic toxicity, before becoming suitable for in vivo tests (*** $p < 0.005$).

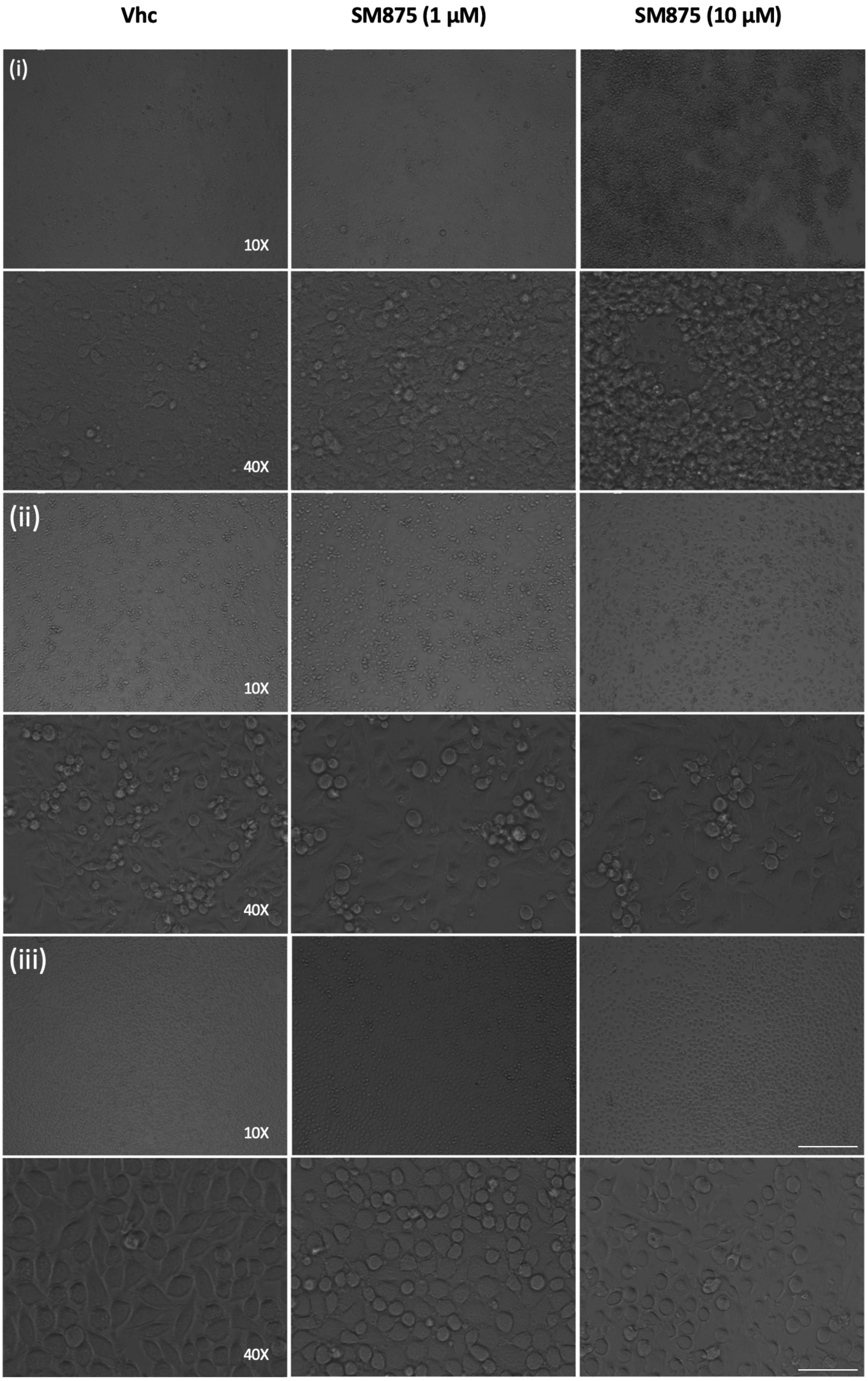

Figure S8: Phase contrast images of cells treated with SM875. The panel shows images of HEK293 (i), ZR-75 (ii) and L929 fibroblasts (iii) upon treatment with SM875 (1 or 10 µM) or vehicle (0.1% DMSO) control. Images were taken at the same incubation time and concentrations employed in the cell viability assay. Scale bars are 400 µm for 10X and 100 µm for 40X.

(i)

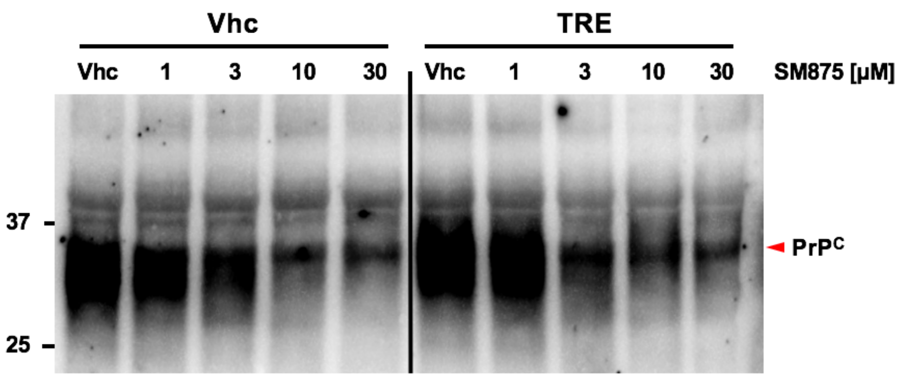

(ii)

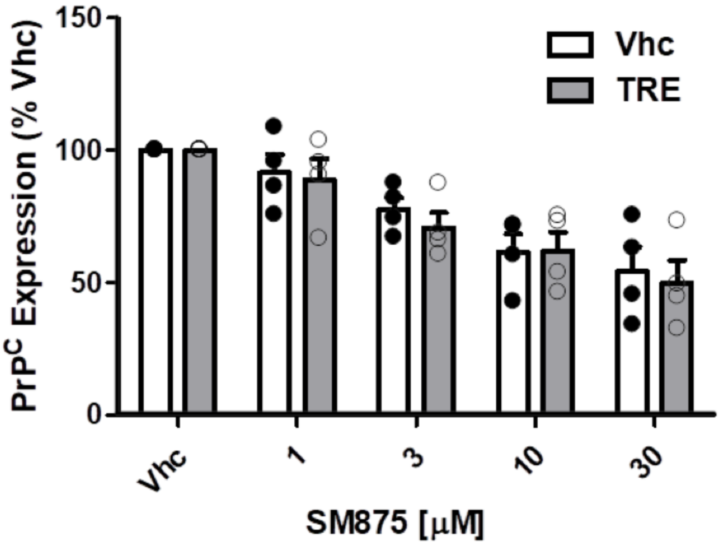

Figure S9: Stimulation of autophagy does not alter the PrP-lowering effects of SM875. Since inhibition of autophagy-mediated lysosomal degradation rescued the decrease of PrP induced by SM875, we sought also to test whether stimulating this pathway would alter the activity of the compound. Thus, we treated ZR-75 cells with different concentrations of SM875 (indicated) or vehicle (DMSO, volume equivalent), in the presence or absence of autophagy inducer TRE (100 µM) and detected PrP by western blotting (i). Signals were detected by probing membrane blots with anti-PrP antibody (D18). We observed that SM875 lowers the levels of full-length PrP (indicated by the red arrow) in a dose-dependent fashion independently from the presence or absence of TRE; (ii) the graphs show the densitometric quantification of full-length PrP from independent replicates (n ± 4). Each signal was normalized on the corresponding total protein lane (detected by UV) and expressed as the percentage of vehicle (Vhc)-treated controls.

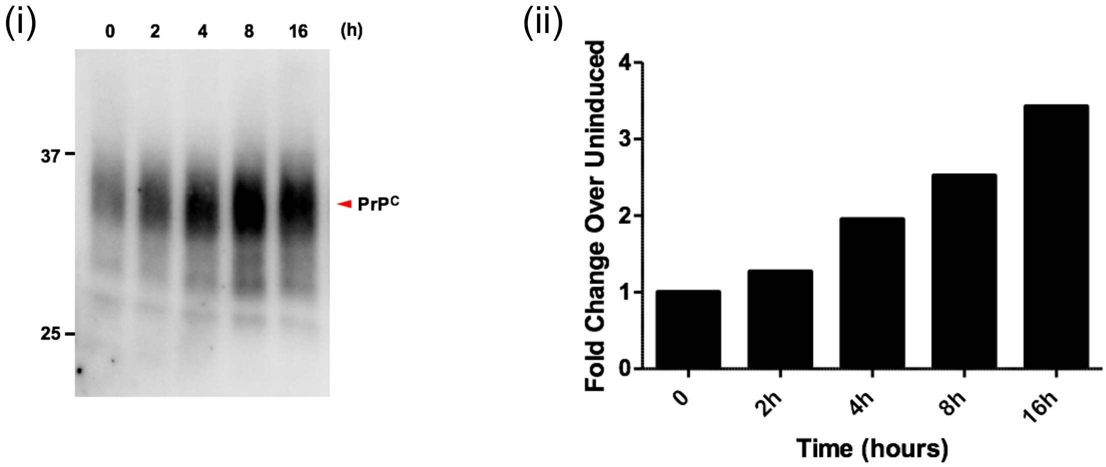

Figure S10: Expression of PrP in doxycycline-inducible RK13 cells. (i) The expression of PrP upon induction with doxycycline (0.01 mg/mL) was evaluated by western blotting over 16 h. Signals were detected by probing membrane blots with anti-PrP antibody (D18); (ii) The graph shows the densitometric quantification of full-length PrP, obtained by normalizing each signal on the corresponding total protein lane (detected by UV) and expressed as fold change of vehicle-treated control.

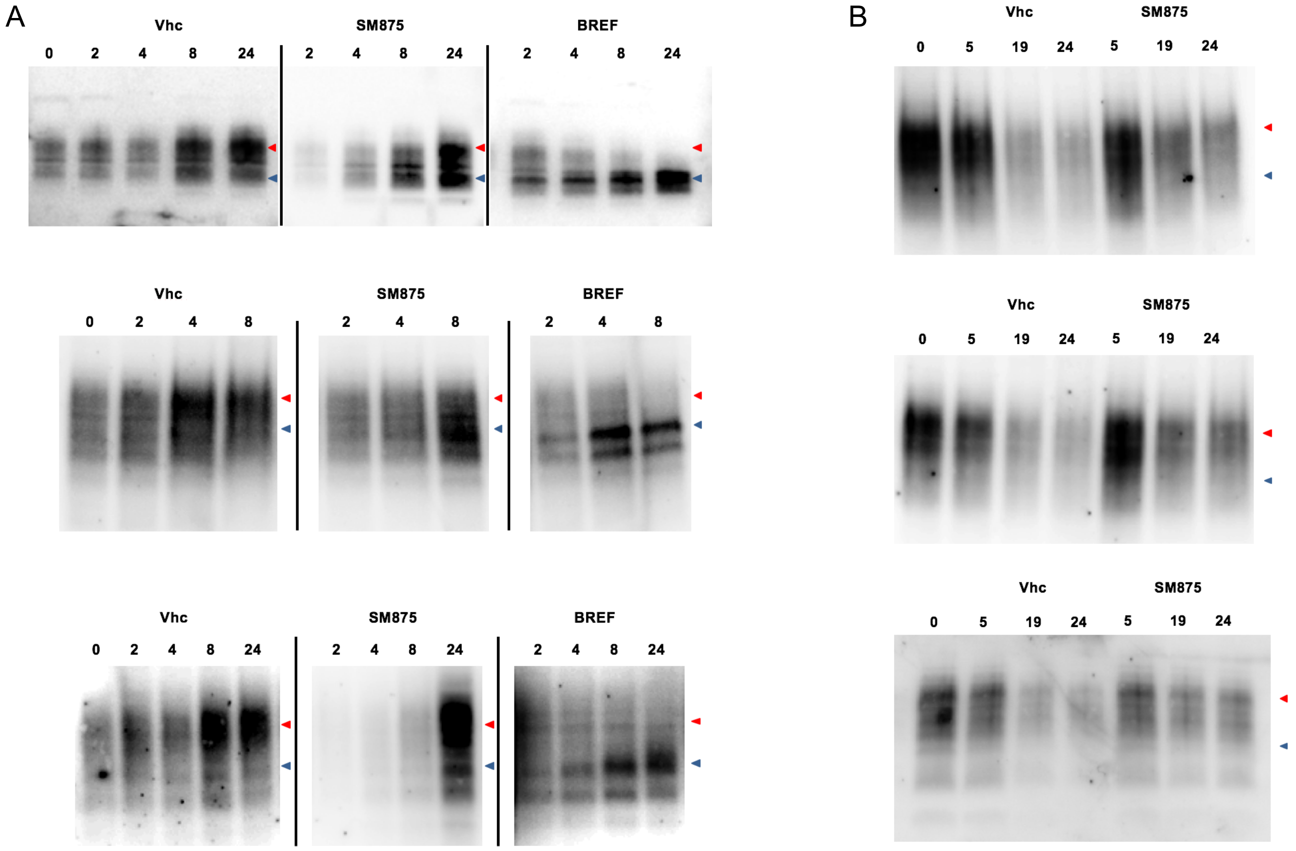

Figure S11: Replicates of experiments presented in figure 5. A. Individual replicates of experiments shown in Figure 5B, panel (i). **B**. Individual replicates of experiments presented in Figure 5B, panel (ii). Signals were detected by probing membrane blots with anti-PrP antibody (D18).

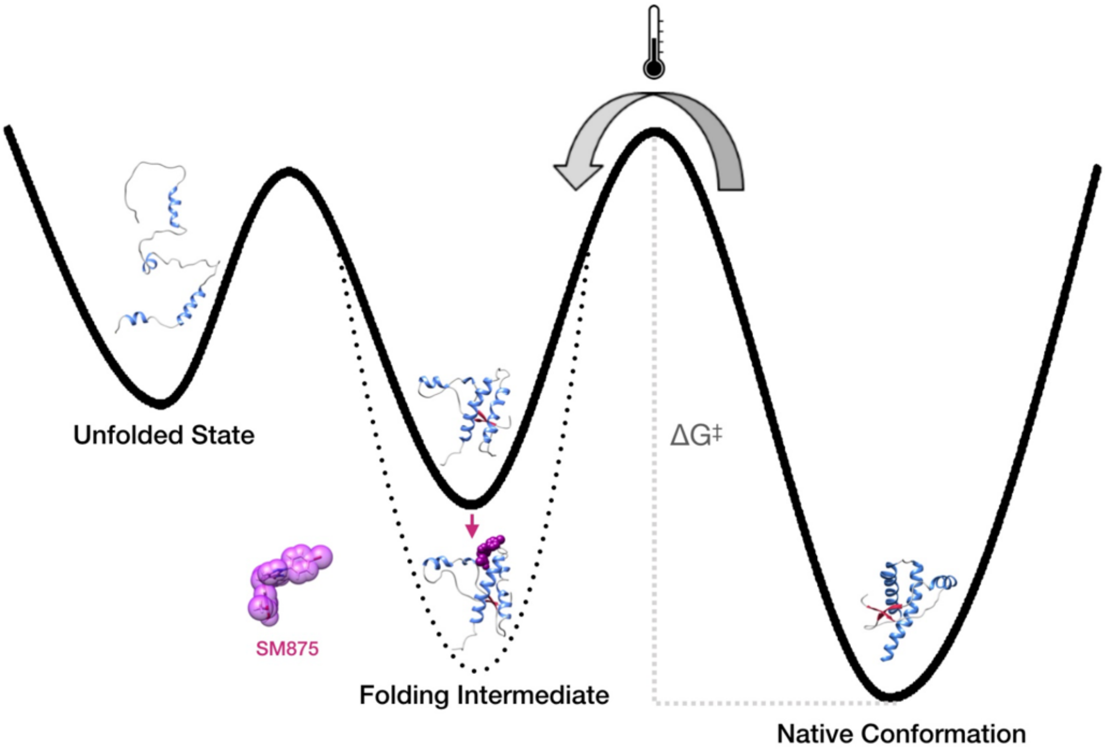

Figure S12: Scheme of the experimental layout used to try co-crystallizing the PrP folding intermediate bound to SM875. In an attempt to structurally characterize the PrP folding intermediate identified in-silico, we designed a novel experimental paradigm to induce the appearance of alternative PrP conformers, separated from the native state by a relevant energy barrier ($\Delta G^{\ddagger}$). This goal was tentatively achieved by the partial, thermal unfolding of mouse recombinant PrP (111-230, 800 µM; temperature raised to 45 °C; the reported melting temperature of PrP is approximately 65 °C[64], and then adding compound SM875.

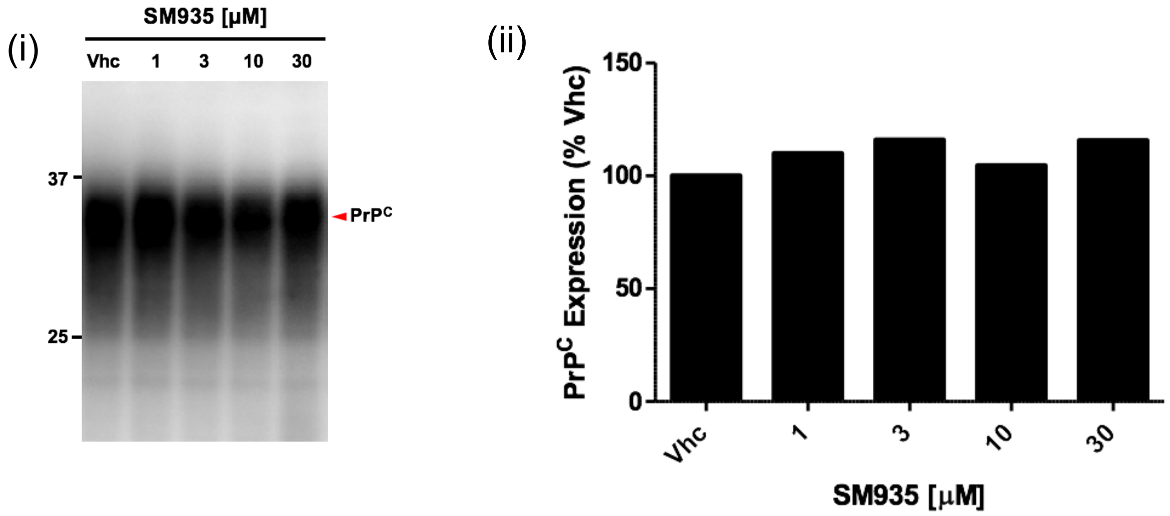

Figure S13: Lack of PrP-lowering effect for compound SM935. HEK293 cells stably transfected with mouse PrP were exposed to different concentrations of SM935 (indicated) or vehicle (Vhc, DMSO, volume equivalent) for 48 h, lysed, and analyzed by western blotting (i). Signals were detected by using a specific anti-PrP (D18). Red arrow indicates the expected sizes of fully glycosylated PrP. The compound shows no effects on the expression of PrP, even at the highest concentration tested (30 µM); (ii) the graph shows the densitometric quantification of the levels of full-length PrP from two independent replicates (n = 2). Each signal was normalized on the corresponding total protein lane (detected by UV) and expressed as the percentage of the level in Vhc-treated controls.

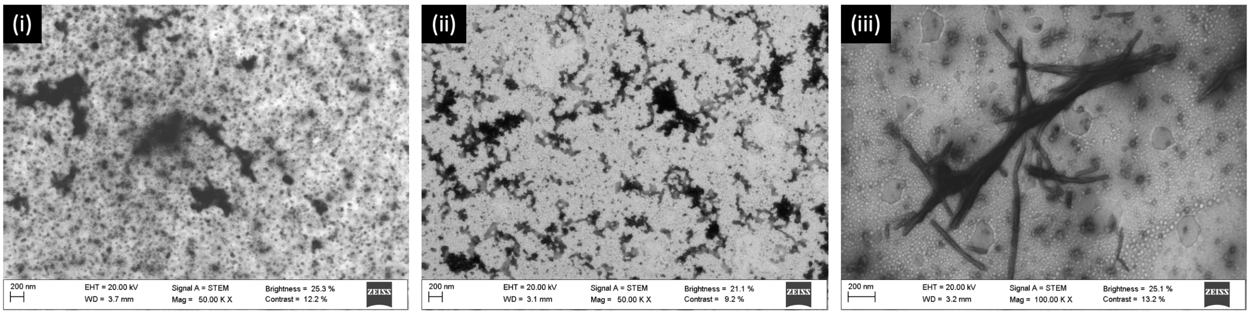

Figure S14: Characterization of SM875-induced PrP aggregates by FESEM. FESEM micrographs in panels (i) and (ii) show aggregates of human recombinant PrP (23-231) induced upon adding compound SM875 and subjecting to mild thermal unfolding (55 °C). Differently from PrP amyloids prepared as previously described[65] (iii), SM875 induced the formation of aggregates appearing as a multitude of dots of stained material of different sizes and shapes (∼10 nm diameter) as well as much larger clumps (i). Sonication did not affect the morphology of these aggregates, leading only to breakage of the larger structures (ii).

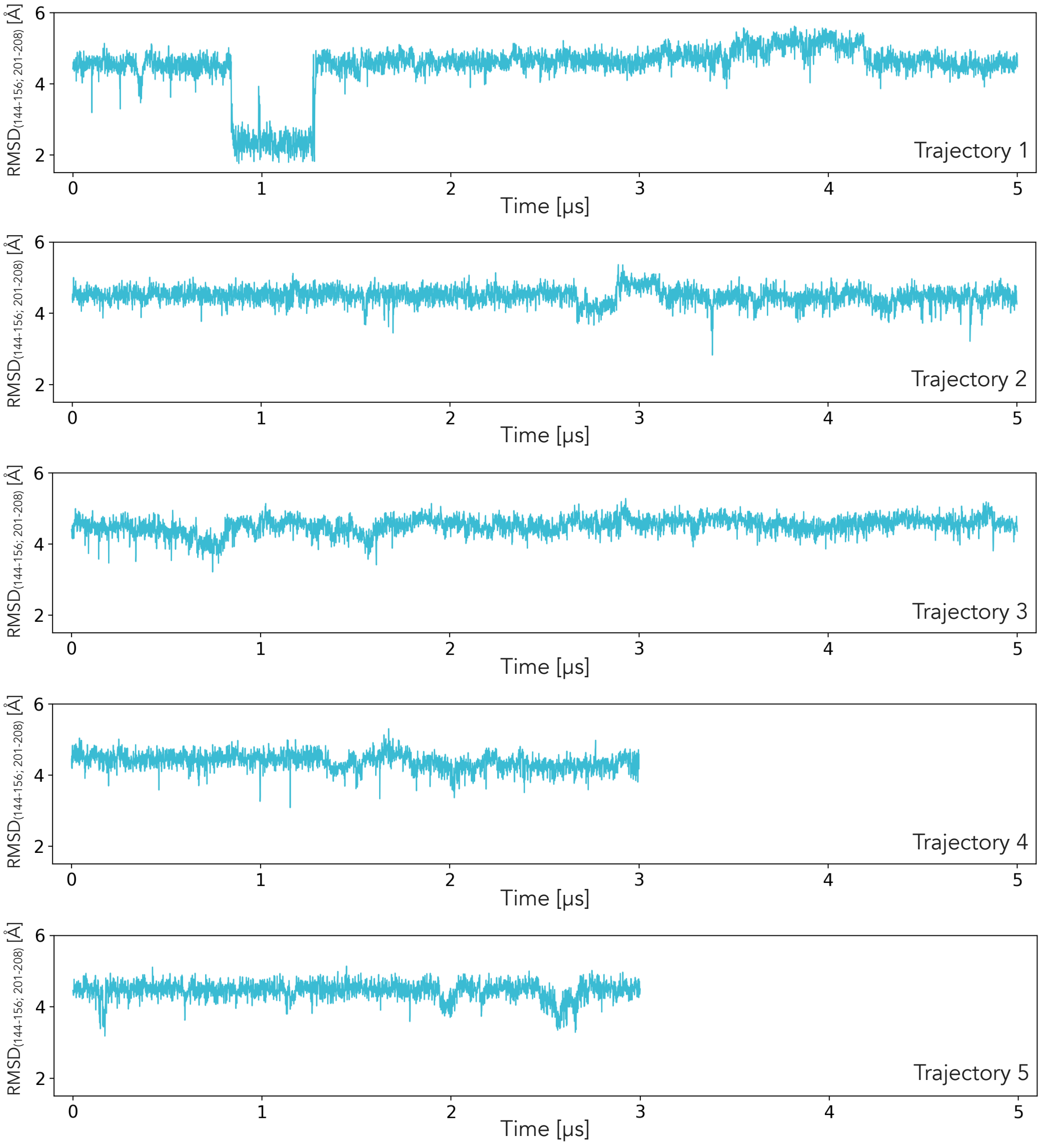

Figure S15: Plain MD simulations of PrP. Native PrP (residues 125-228, PDB 1QLX) was subjected to three replicas of 5 us and two replicas of 3 us each at 310 K. The RMSD of the contact region between helix 1 and helix 3 (residues 144-156 and 201-208) was computed with respect to the structure of the identified PrP folding intermediate.

Figure S16. Uncropped western blot images.

Western blot images are reported as originally acquired using the ChemiDoc XRS Touch Imaging System (Bio-Rad). The corresponding figure in the main manuscript is reported below each image.

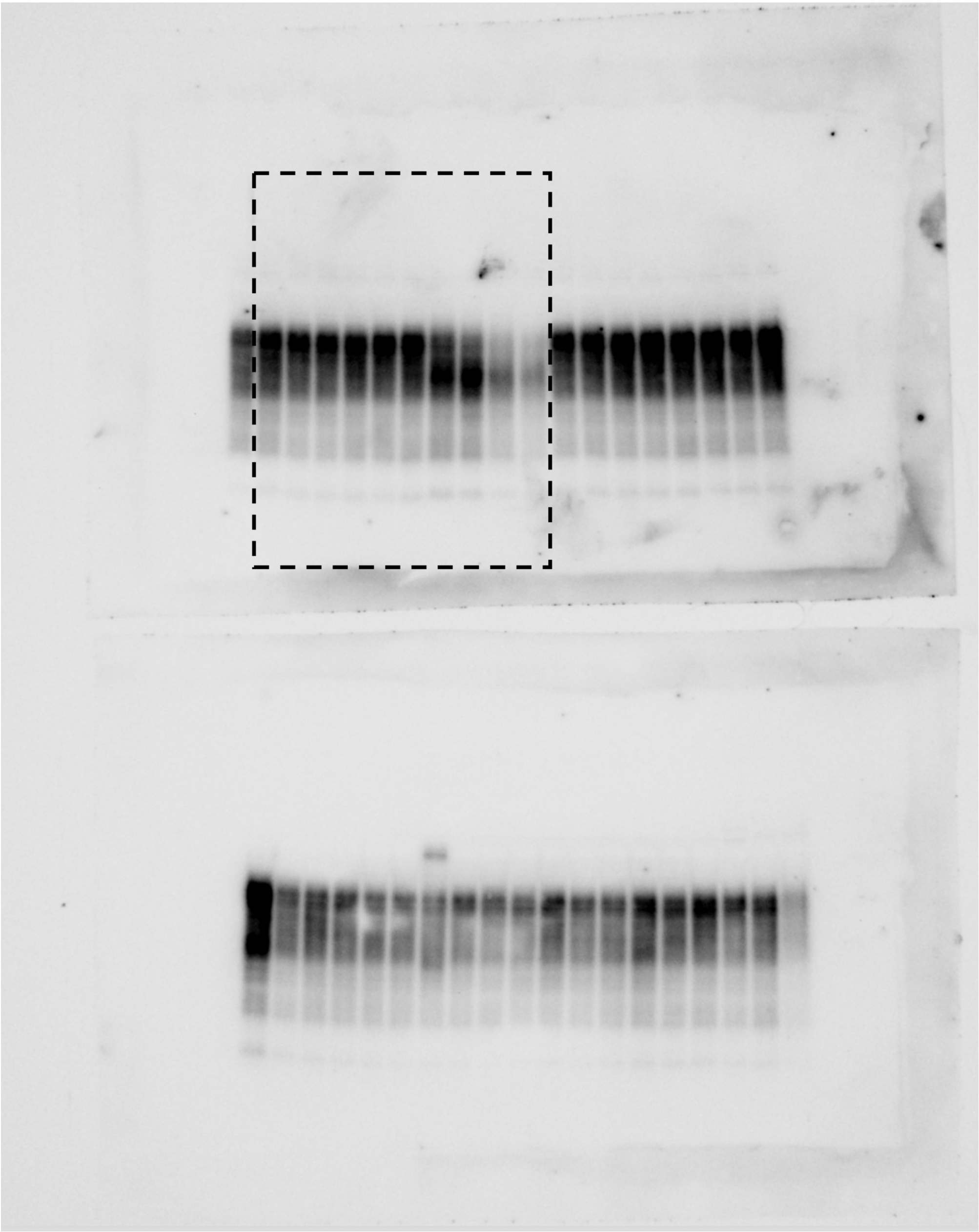

Figure S16: Figure 2a (i)

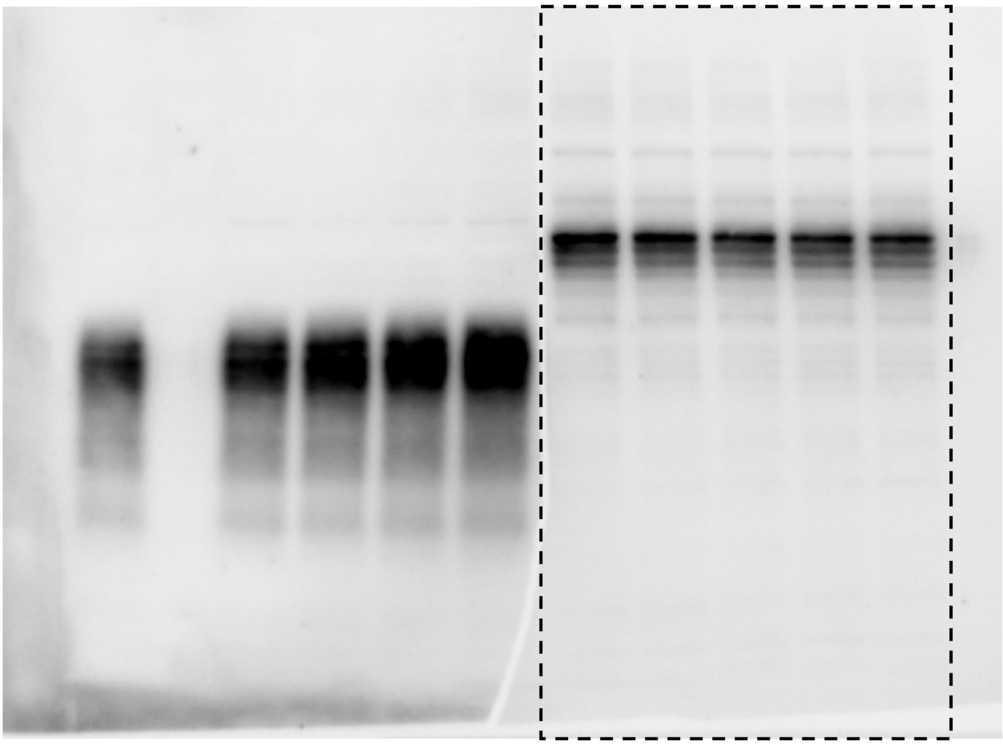

Figure S16: Figure 2a (ii)

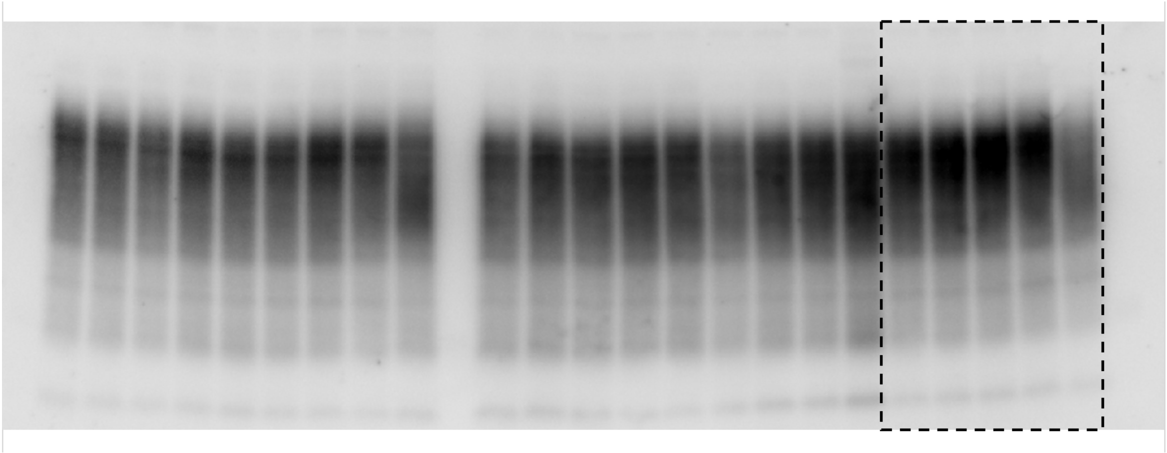

Figure S16: Figure 2b (i)

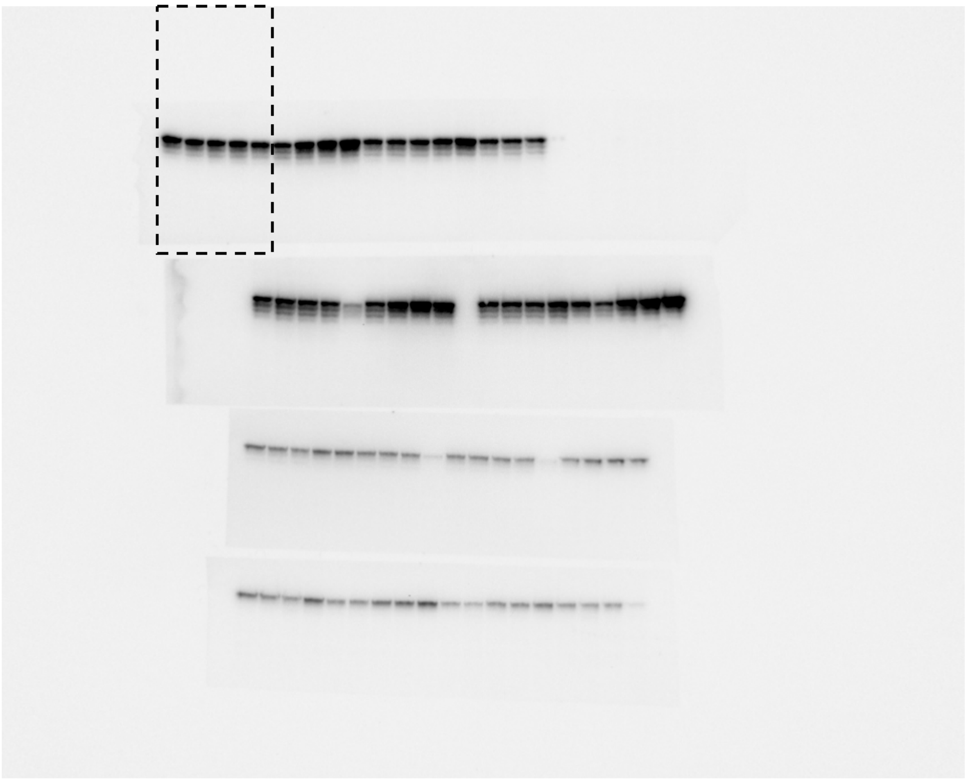

Figure S16: Figure 2b (ii)

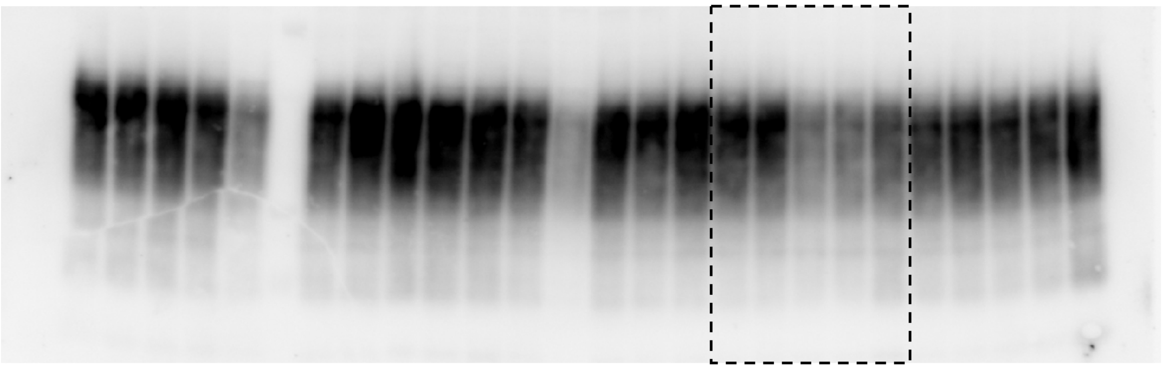

Figure S16: Figure 2c (i)

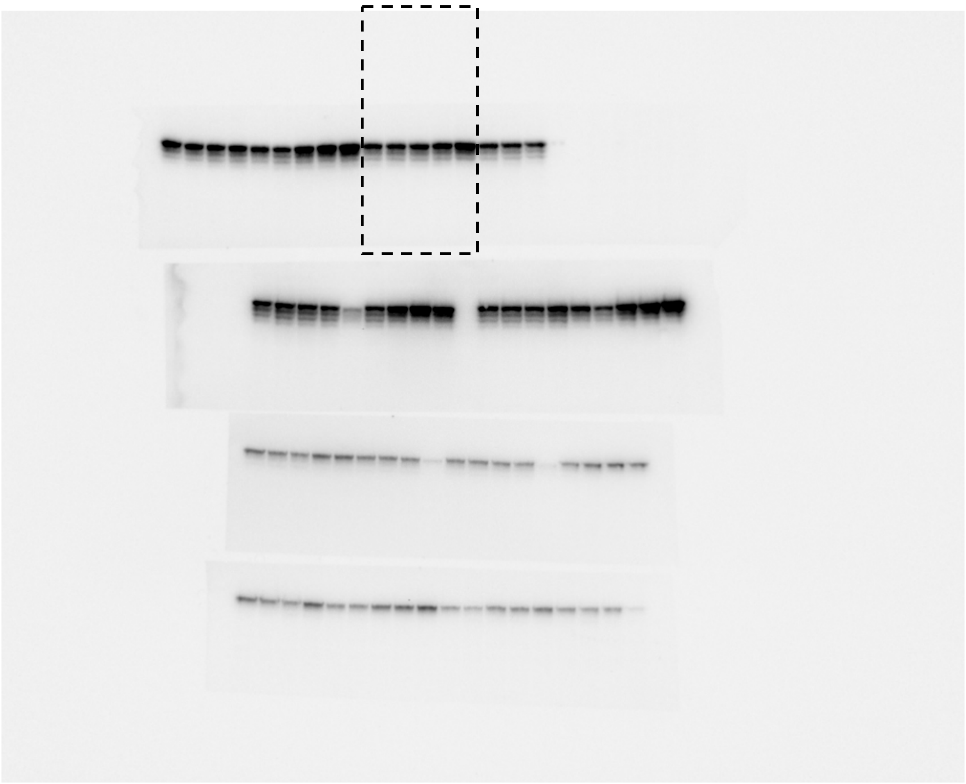

Figure S16: Figure 2c (ii)

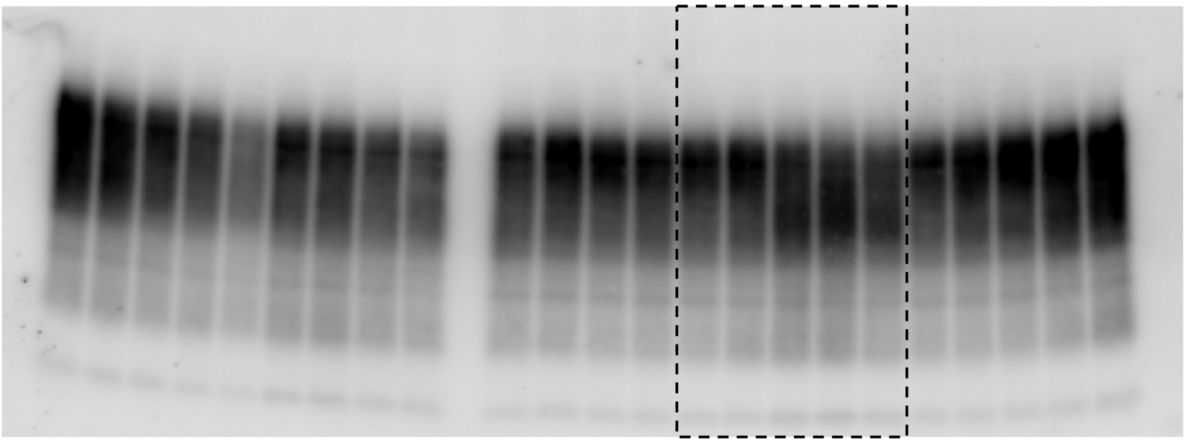

Figure S16: **Figure 2d (i)**

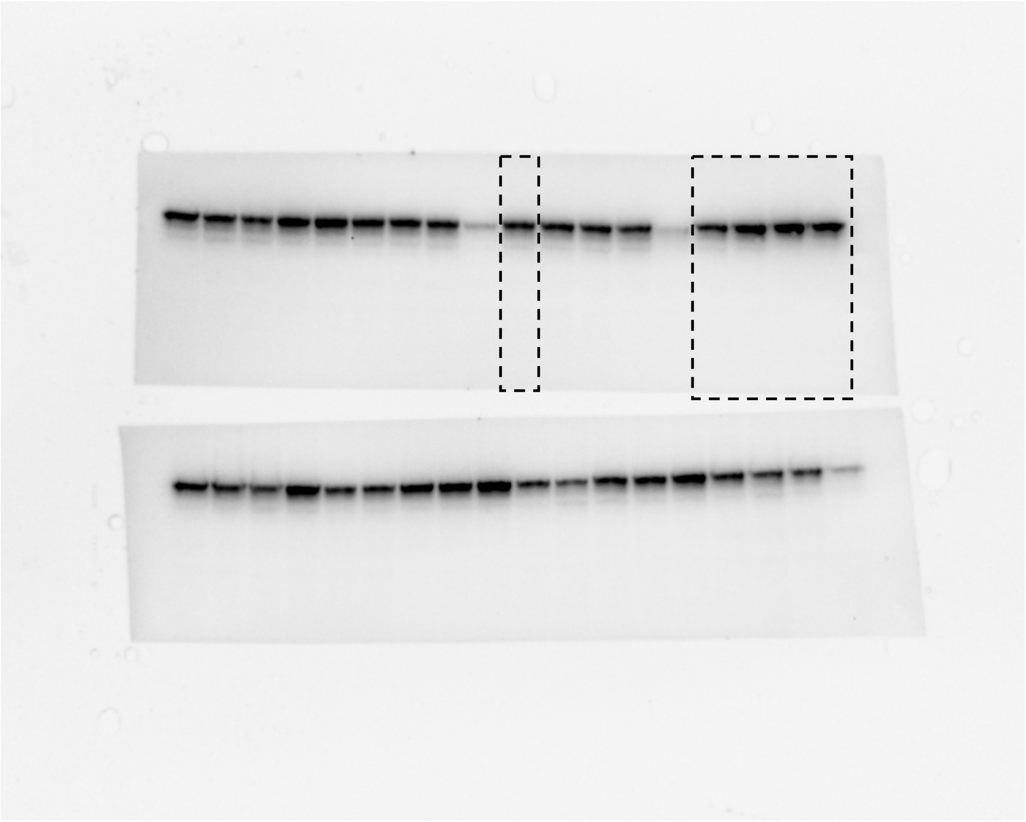

Figure S16: **Figure 2d (ii)**

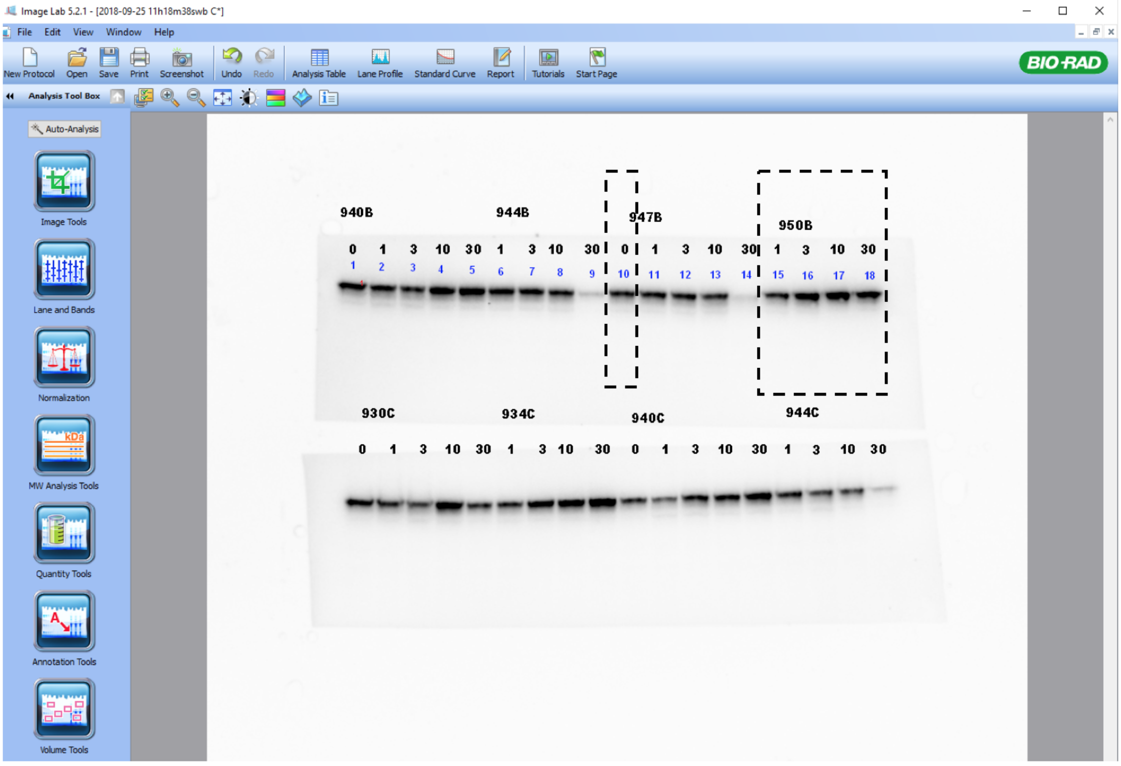

Figure S16: Figure 2d (ii) Layout Numbering

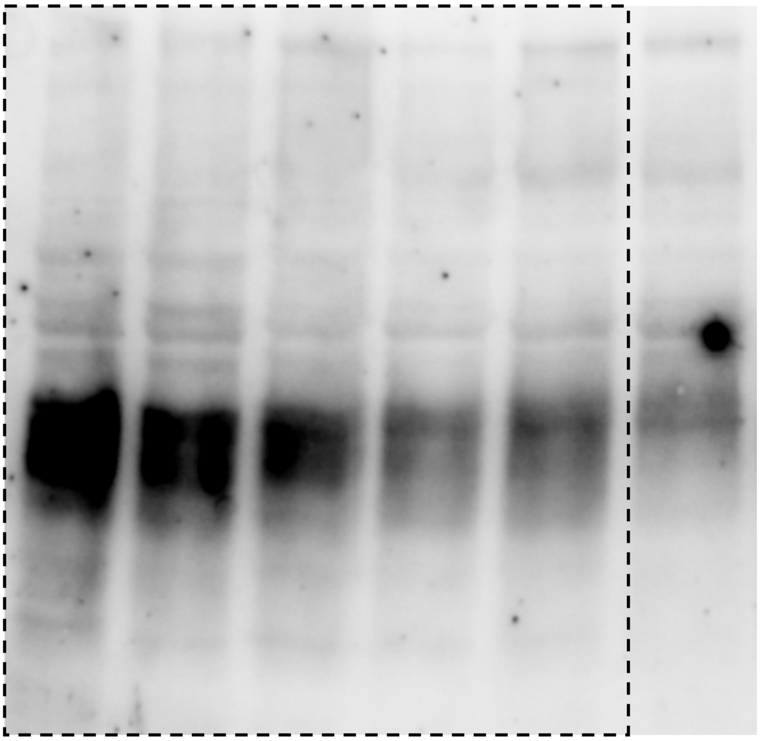

Figure S16: **Figure 3a (i)**

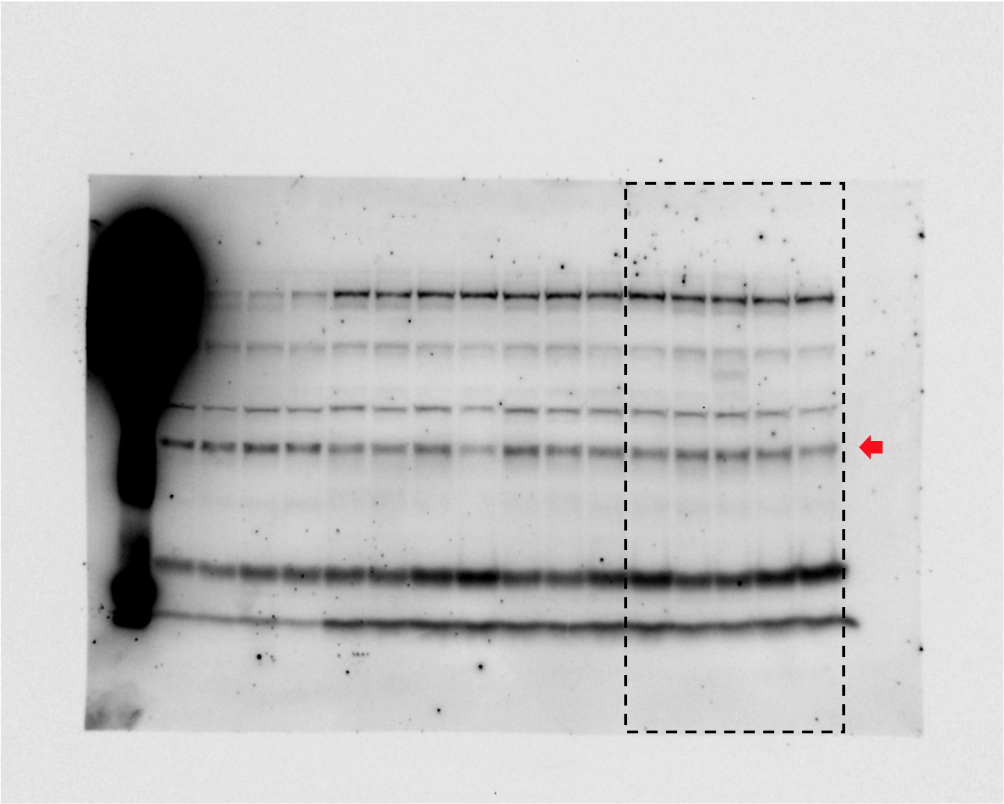

Figure S16: **Figure 3a (ii)**

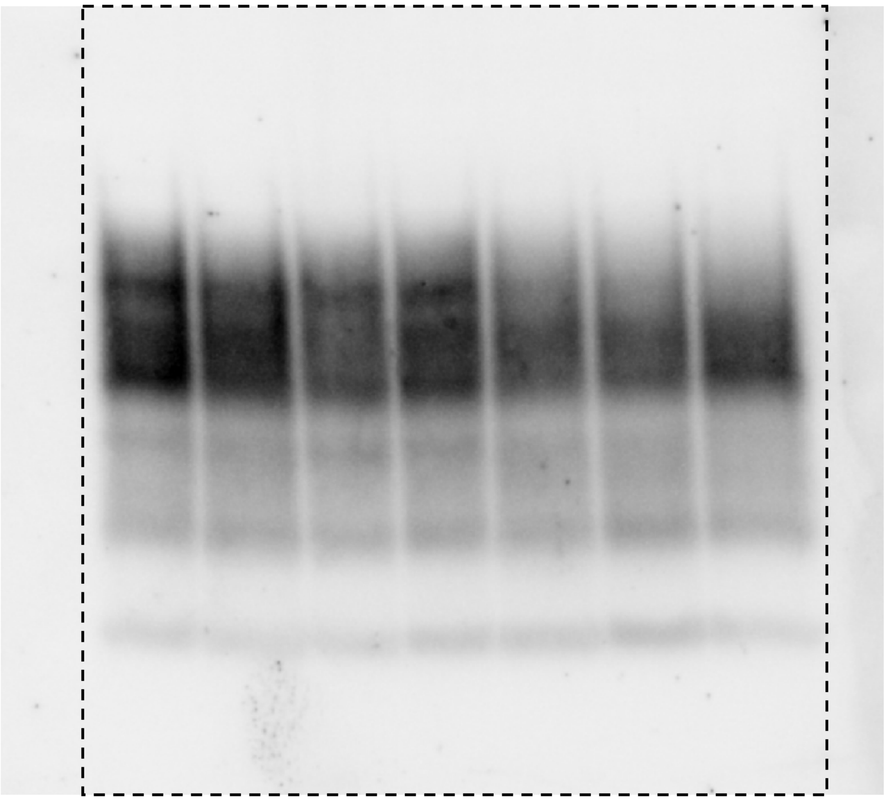

Figure S16: **Figure 3b (i)**

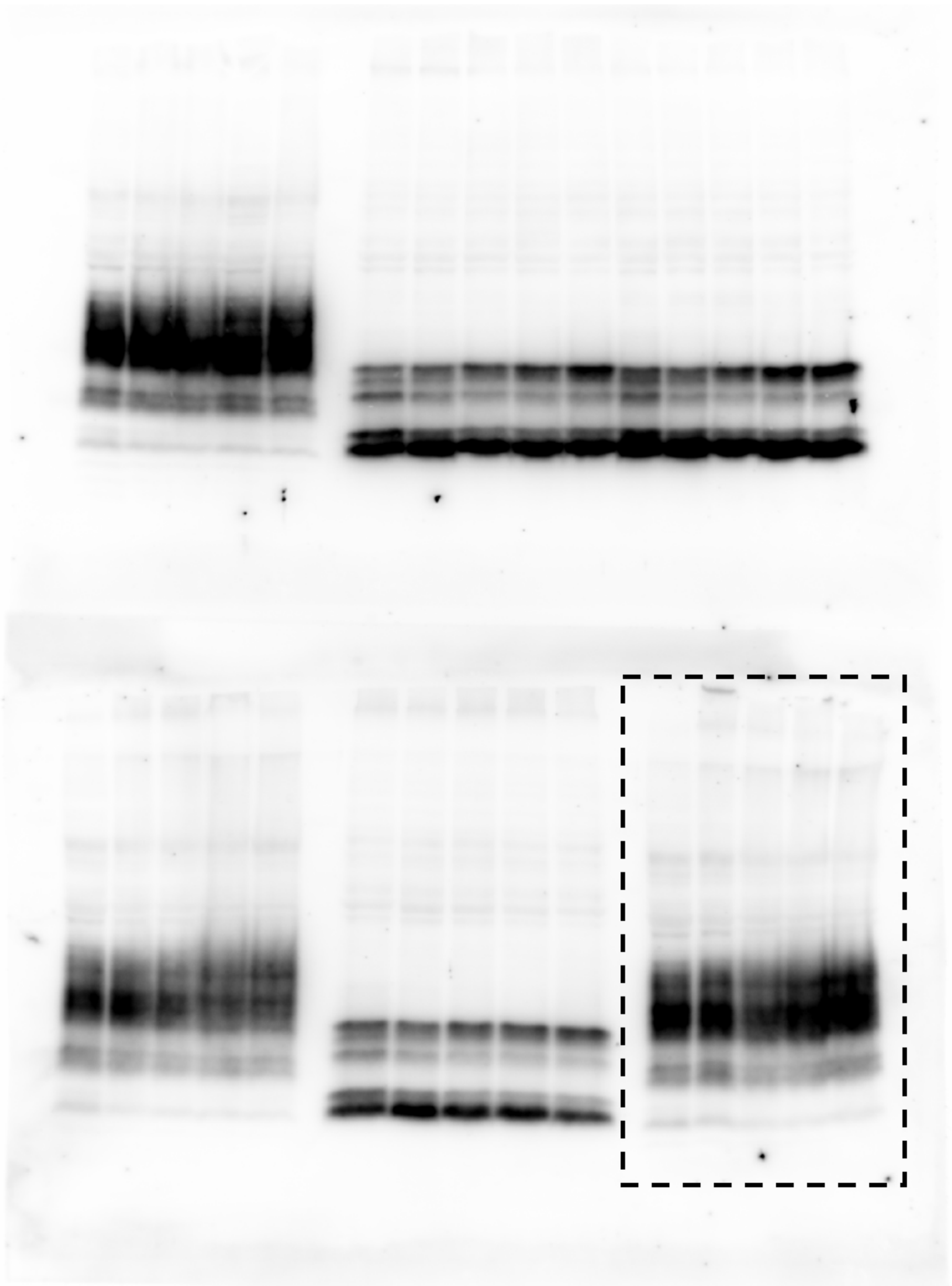

Figure S16: **Figure 3c (i)**

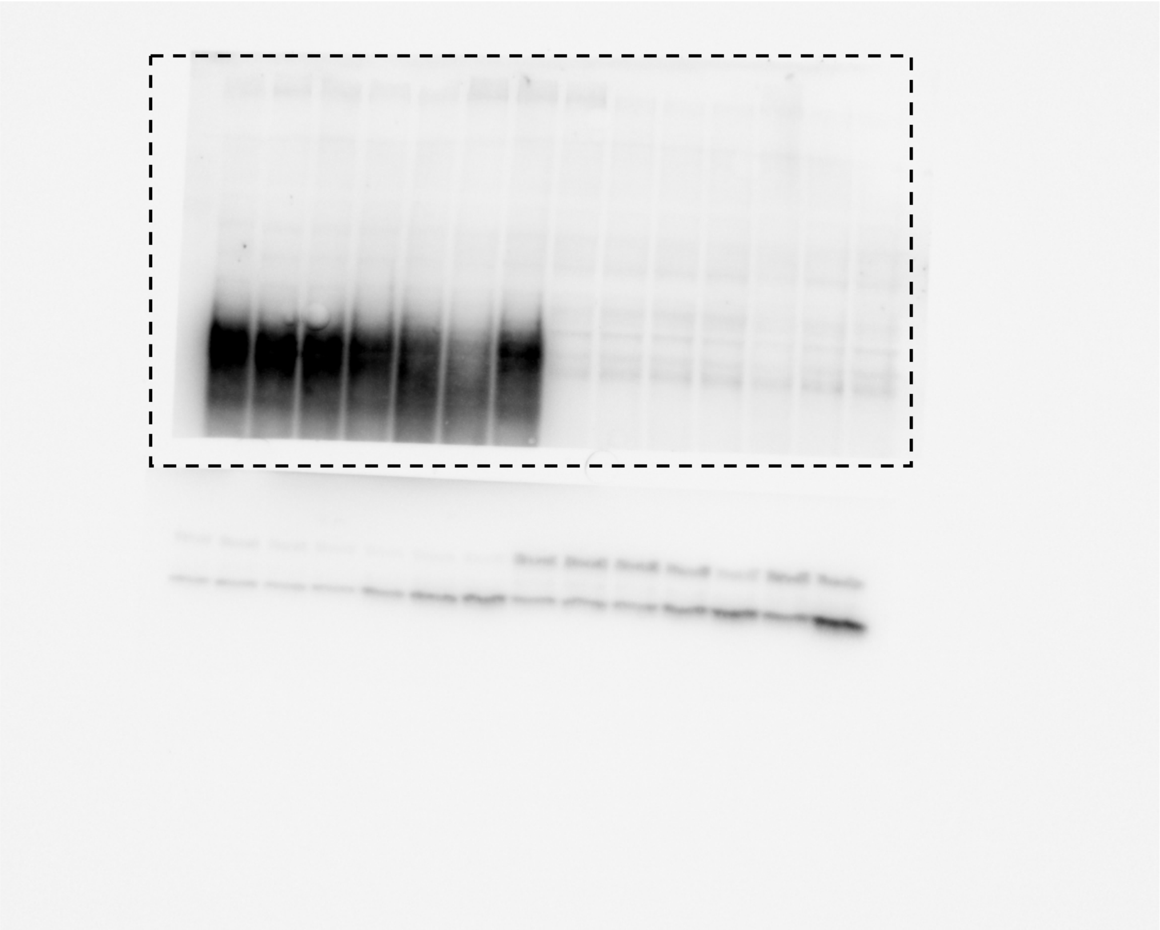

Figure S16: **Figure 4a (i) - PrP^{C}**

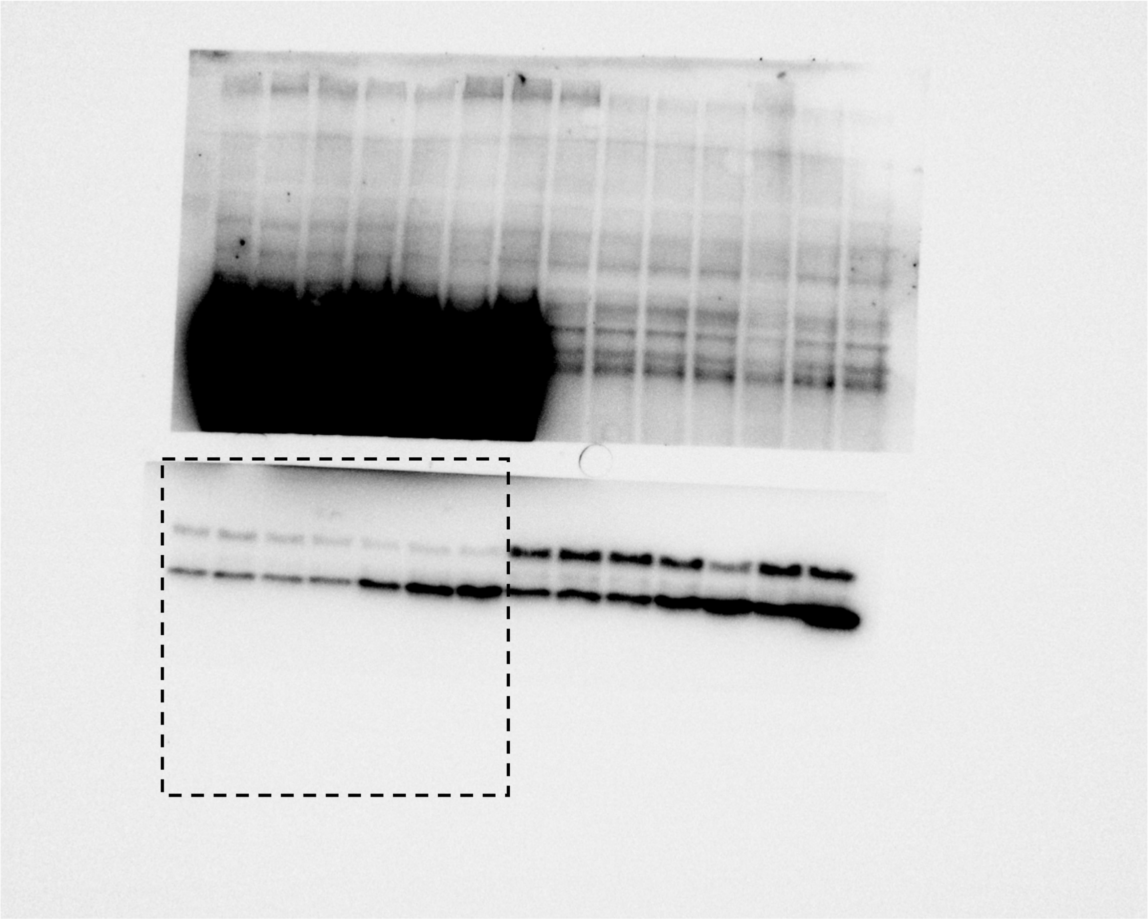

Figure S16: Figure 4a (i) - LC3

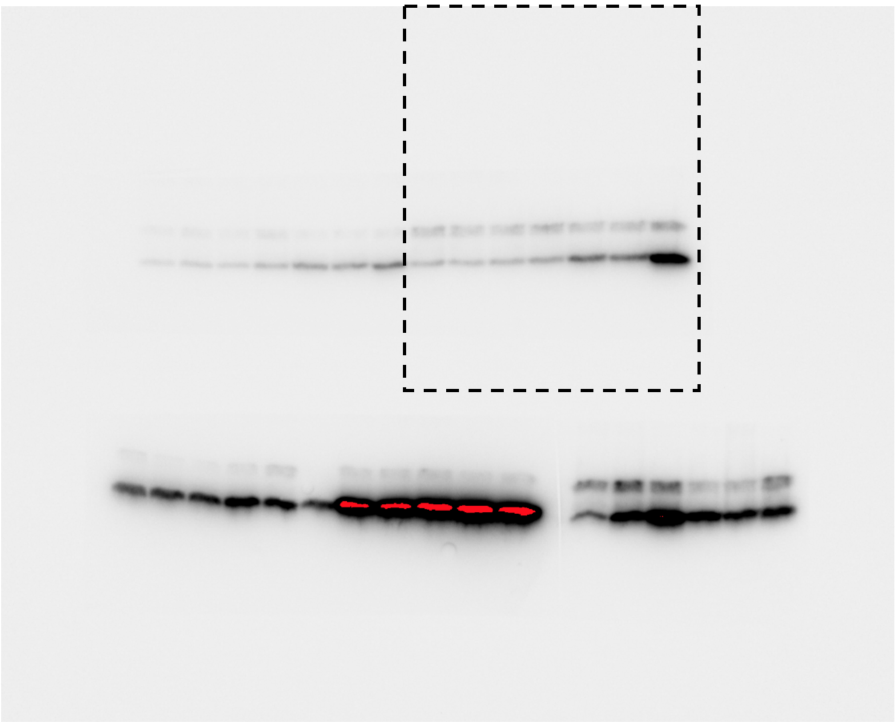

Figure S16: Figure 4a (i) - LC3

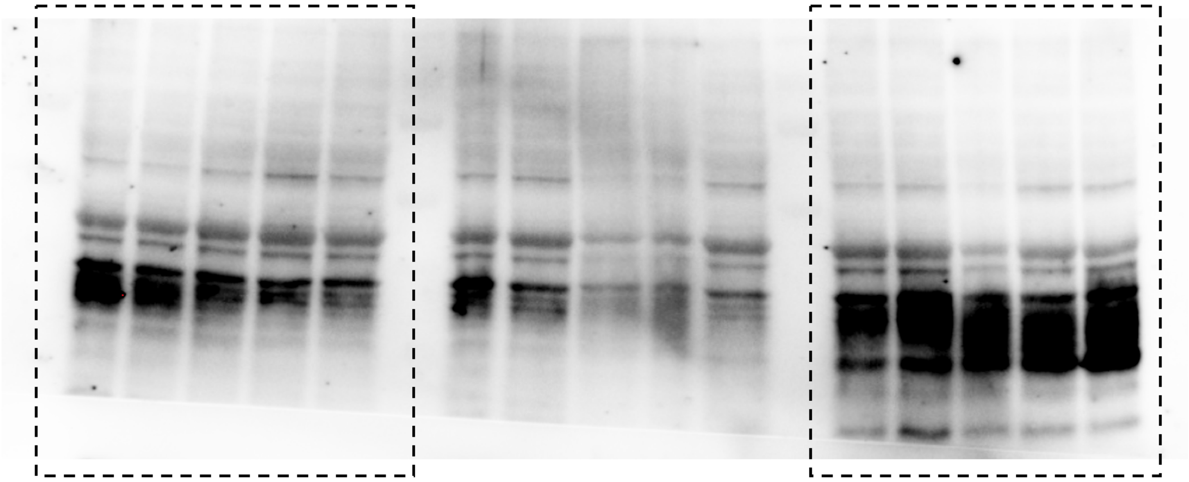

Figure S16: **Figure 4b (i)**

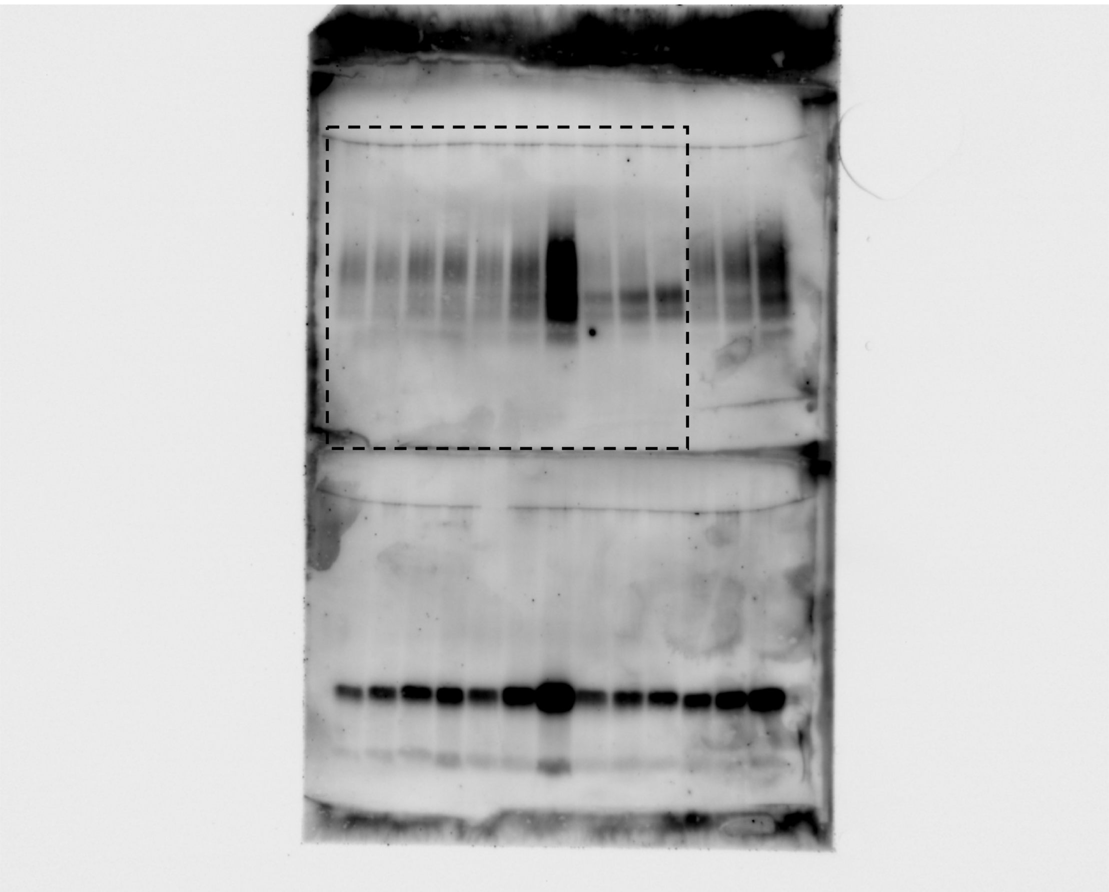

Figure S16: Figure 5b (i)

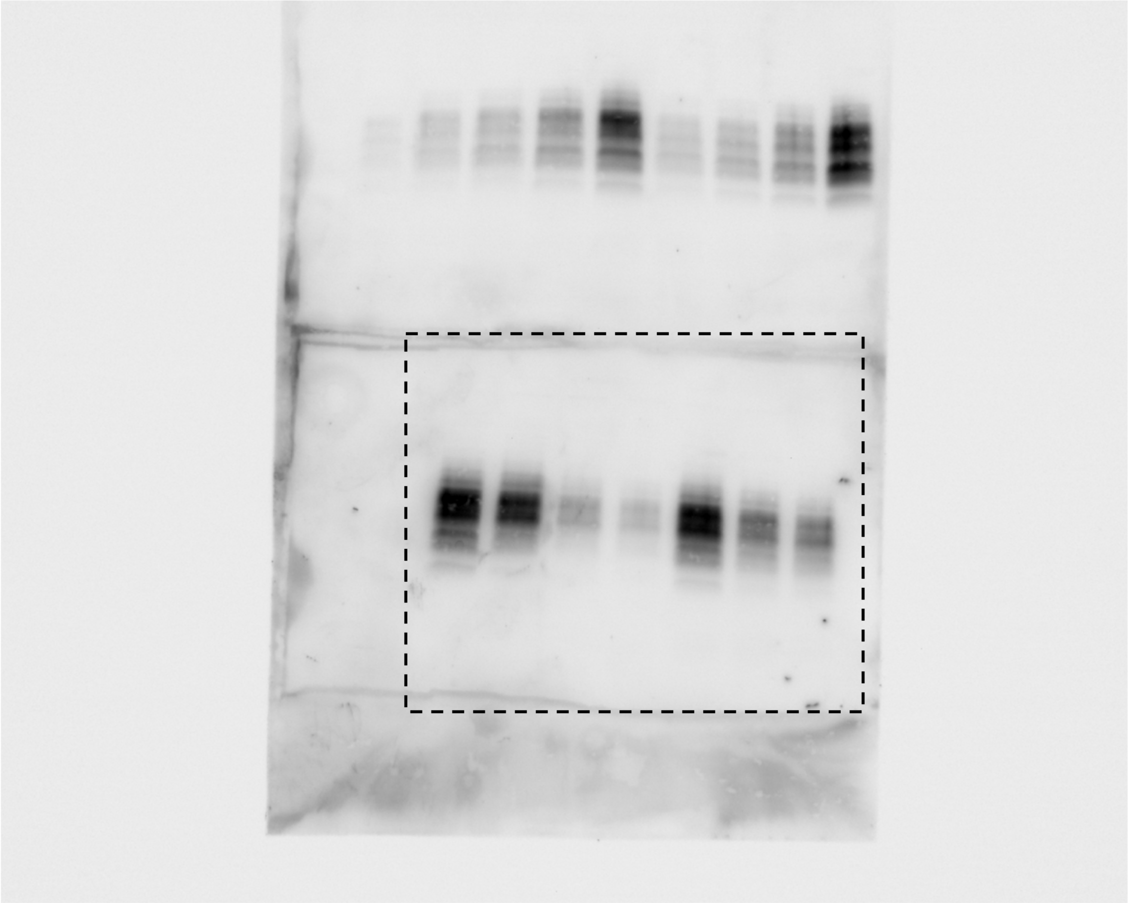

Figure S16: **Figure 5b (ii)**

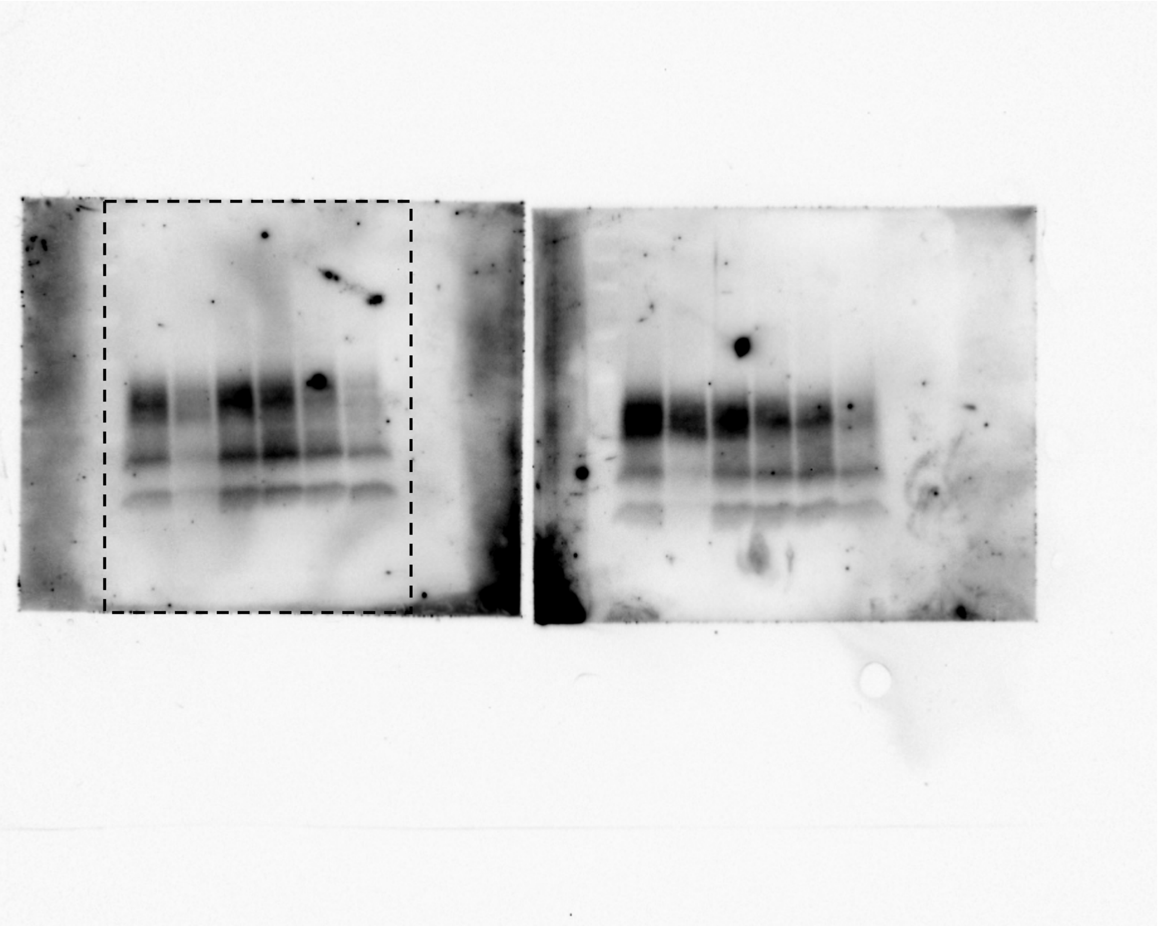

Figure S16: **Figure 6b (i)**

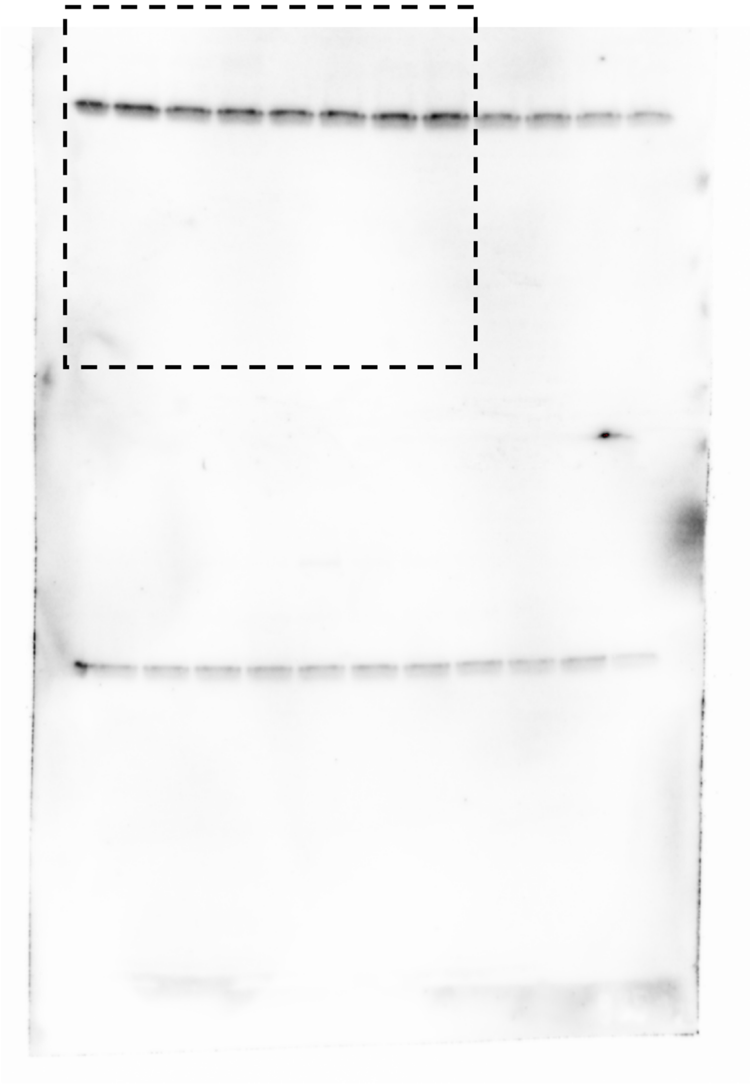

Figure S16: **Figure 7a (i) - SM935**

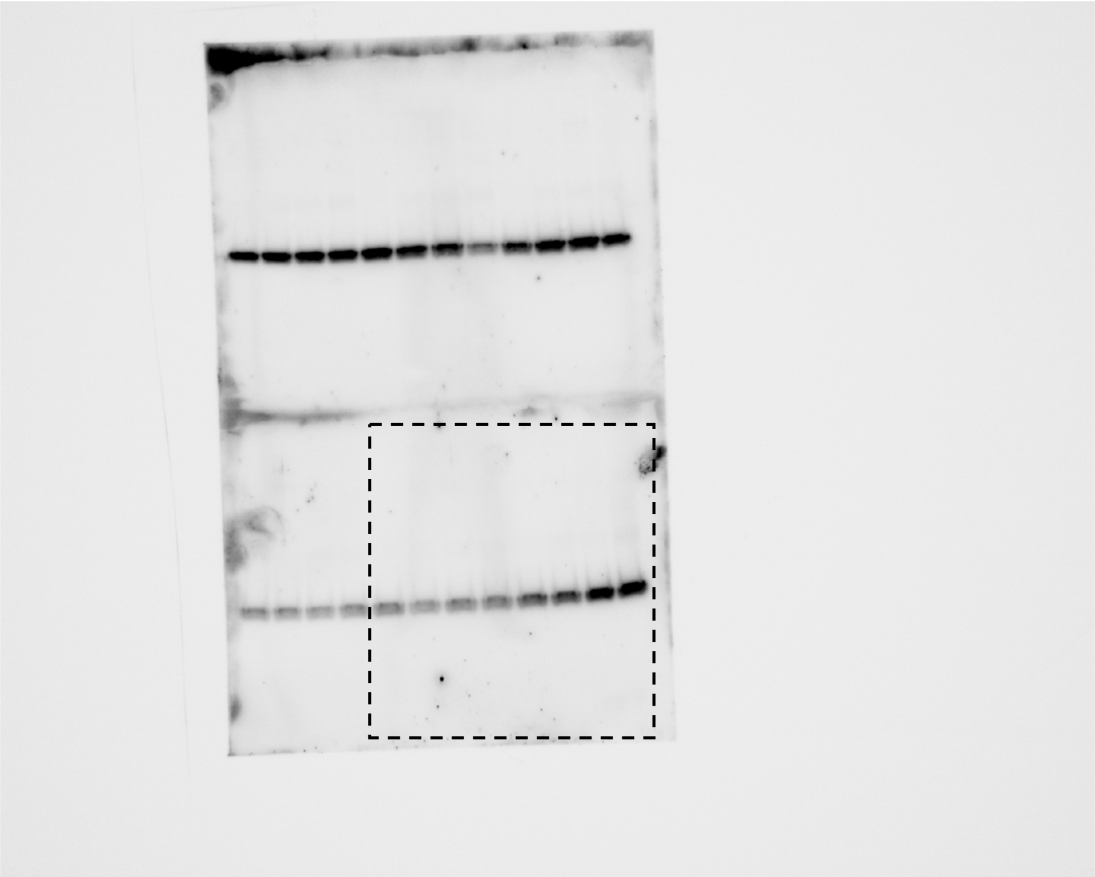

Figure S16: Figure 7a (i) - SM875

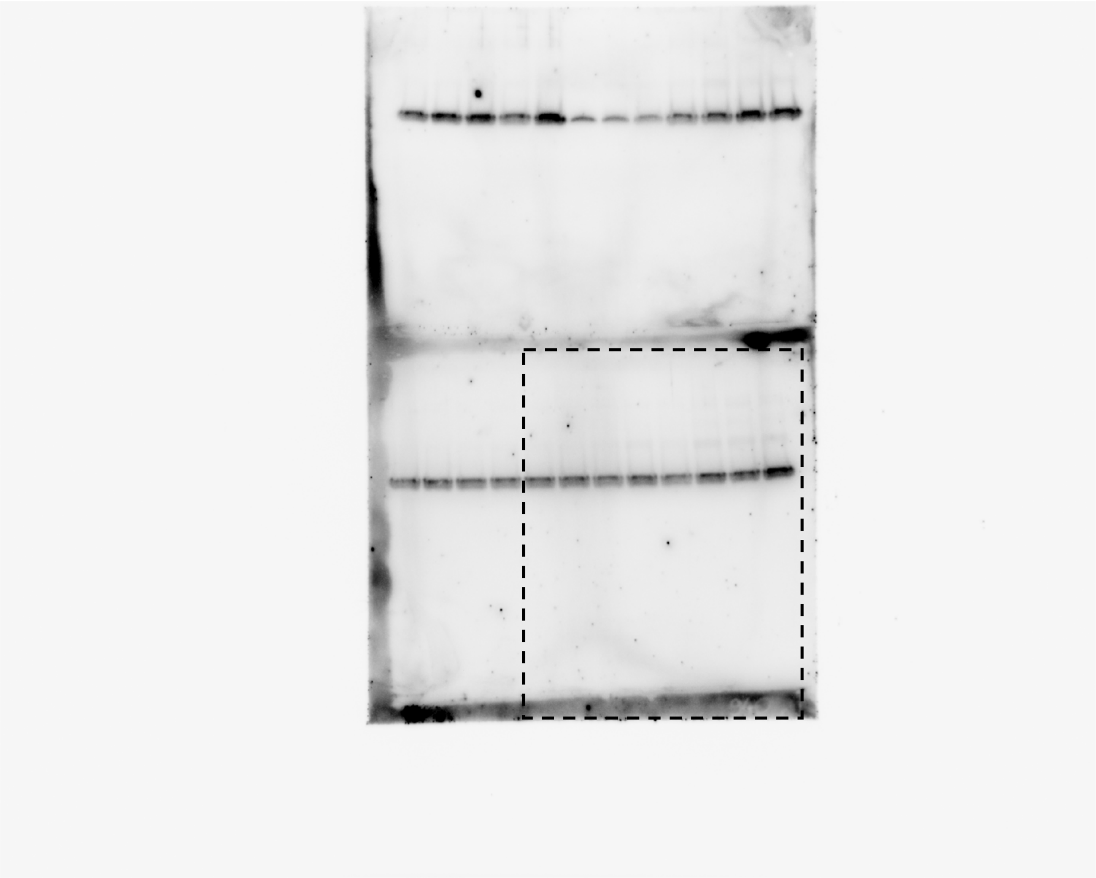

Figure S16: **Figure 7a (i) - SM940**

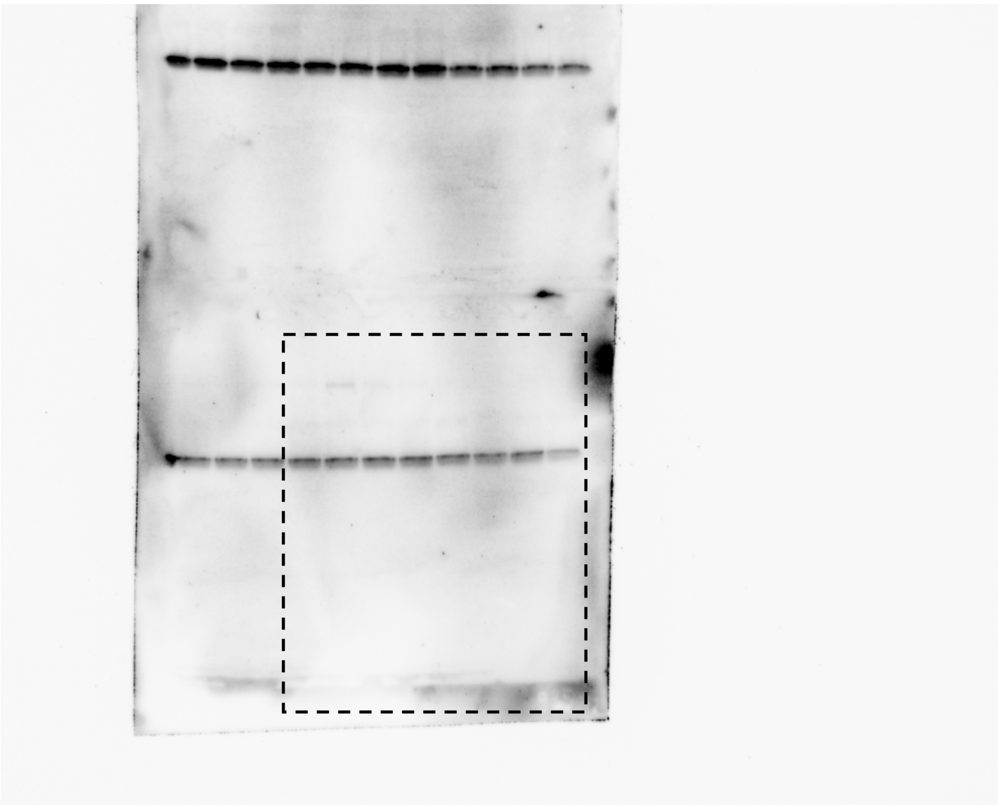

Figure S16: **Figure 7a (i) - Blank**

Supplementary Tables

(i)

Descriptor	Threshold	PrP I Pocket
SiteScore[a]	≥ 0.8	0.95
DScore[a]	≥ 0.9	0.99
Exposure[a]	≤ 0.5	0.60
Enclosure[a]	≥ 0.7	0.66
Balance[a]	≥ 1.0	0.65
Volume[b]	≥ 300 Å^3	468.6 Å^3
Depth[b]	≥ 10 Å	10.4 Å
SimpleScore[b]	≥ 0.5	0.38
DrugScore[b]	≥ 0.5	0.23
Descriptors computed using SiteMap[a] and DogSiteScorer[b]		

(ii)

Descriptor	Threshold	Representative MD Conformations of PrP Intermediate Pocket									
		1	2	3	4	5	6	7	8	9	10
SiteScore[a]	≥ 0.8	n/a	0.75	0.79	0.74	0.73	0.62	0.84	0.94	0.95	1.00
DScore[a]	≥ 0.9	n/a	0.73	0.75	0.71	0.74	0.56	0.85	0.94	1.00	1.06
Exposure[a]	≤ 0.5	n/a	0.75	0.51	0.61	0.72	0.66	0.57	0.47	0.59	0.68
Enclosure[a]	≥ 0.7	n/a	0.65	0.74	0.65	0.56	0.59	0.65	0.75	0.64	0.63
Balance[a]	≥ 1.0	n/a	0.58	1.69	1.43	0.90	0.14	1.76	1.58	1.89	0.82
Volume[b]	≥ 300 Å^3	169.8	154.4	219.5	262.5	210.1	62.0	283.5	313.5	3904.2	284.2
Depth[b]	≥ 10 Å	9.76	10.96	11.13	13.54	11.15	9.00	14.44	13.59	15.56	12.66
SimpleScore[b]	≥ 0.5	0.18	0.34	0.32	0.41	0.39	0.37	0.36	0.52	0.50	0.42
DrugScore[b]	≥ 0.5	0.07	0.25	0.25	0.34	0.29	0.31	0.36	0.53	0.48	0.35
Descriptors computed using SiteMap[a] and DogSiteScorer[b]											

Table S1: Druggability descriptors of the pocket in the PrP folding intermediate. The tables illustrate the computed SiteMap and DogSiteScorer descriptors for the pocket conformation before (i) or after (ii) MD simulations. The MD simulation gave rise to ten different representative conformations of the pocket. The analysis highlights the refined site in the MD conformation n.8, which possesses the desired thresholds for a well-defined, solvent-accessible druggable ligand-binding site. Noteworthy, this pocket was not present in the native form of PrP.

Compound ID	SM code	K_{iHYDE}
**ASN19380133**	875	40275 nM > Ki > 405 nM
**BAS00670252**	923	17266 nM > Ki > 174 nM
**BAS01809156**	924	468 nM > Ki > 5 nM
**BAS03200180**	925	23544 nM > Ki > 237 nM
**BAS00312802**	926	43535 nM > Ki > 438 nM
**BAS02102991**	927	13673 nM > Ki > 138 nM
**BAS00781325**	929	538 nM > Ki > 5 nM
**BAS00287978**	930	2618 nM > Ki > 26 nM
**BAS01849776**	931	2795 nM > Ki > 28 nM
**BAS00855482**	932	23755 nM > Ki > 239 nM
**BAS01029925**	933	126 nM > Ki > 1 nM
**ASN02751074**	934	5153 nM > Ki > 52 nM
**BAS05541419**	935	7775 nM > Ki > 78 nM
**ASN02241765**	936	6024 nM > Ki > 61 nM
**ASN15755504**	937	2296 nM > Ki > 23 nM
**BAS15232183**	938	5101 nM > Ki > 51 nM
**BAS01095083**	939	20390 nM > Ki > 205 nM
**ASN16356774**	940	6121 nM > Ki > 62 nM
**ASN05397475**	941	2020 nM > Ki > 20 nM
**BAS01195857**	942	1583 nM > Ki > 16 nM
**BAS17126510**	943	26641 nM > Ki > 268 nM
**ASN17325626**	944	5761 nM > Ki > 58 nM
**BAS03374806**	945	19591 nM > Ki > 197 nM
**BAS01515776**	946	20171 nM > Ki > 203 nM
**BAS00382671**	947	40423 nM > Ki > 407 nM
**BAS01990851**	948	15124 nM > Ki > 152 nM
**BAS03321293S**	949	1811 nM > Ki > 18 nM
**BAS01923101**	950	25764 nM > Ki > 259 nM
**BAS01058340**	951	7910 nM > Ki > 80 nM
**BAS09812417**	952	11821 nM > Ki > 119 nM

Table S2: Virtual screening results. The table reports the Asinex ID code, laboratory-internal code (SM code), and predicted HYDE affinity range (K_{iHYDE}) for the 30 virtual hits selected as PrP intermediate ligands. The virtual screening campaign was carried out by means of BioSolveIT tools integrated into an in-house developed KNIME workflow. A funnel-like approach involving the prediction of pharmacodynamics, physicochemical, and ADMET properties as well as molecular diversity and 3D visualization of ligand binding interactions guided the hit selection procedure.

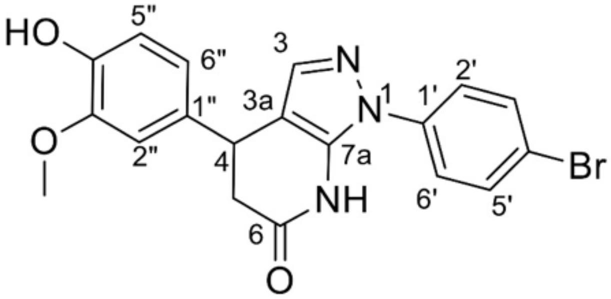

Position	δ_H [ppm] (J [Hz])	δ_C [ppm]	HMBC $^1H \rightarrow ^{13}C$
**3**	7.29 s	138.2	H3 → C3a
**3a**	—	105.9	—
**4**	4.21 dd (9.9, 6.4)	35.5	H4 → C3a, C5, C6, C7a, C1", C2"
**5**	2.93 dd (16.2, 6.4)<br>2.80 dd (16.2, 9.9)	40.9	H5 → C3a, C4, C6, C1"
**6**	—	170.7	—
**7**	8.52 s (NH)	—	H7 → C7a
**7a**	—	136.5	—
**1'**	—	137.3	—
**2', 6'**	7.39 d (8.6)	124.5	H2'/6' → C1', C3'/C5'
**3', 5'**	7.62 d (8.6)	132.9	H3'/5' → C4'
**4'**	—	121.6	—
**1"**	—	133.5	—
**2"**	6.77 s	109.6	H2" → C4, C4"
**3"**	—	146.9	—
**4"**	—	145.0	—
**5"**	6.88 d (8.8)	114.7	H5" → C2", C4"
**6"**	6.76 m	120.0	H6" → C3"
**OCH_3**	3.87 s	55.9	OCH_3 → C3"
**OH**	5.72 vbr s	—	—

Table S3: NMR spectral data of SM875. NMR data obtained in $CDCl_3$ (^{1}H at 400 MHz, ^{13}C at 100 MHz) are reported. Resonances are assigned according to the carbon numbering shown on the chemical structure. In the second column are reported the ^{1}H NMR parameters [δH (J)], in the third column the ^{13}C chemical shifts (δC) and in the fourth column the most relevant long-range heteronuclear correlations (obtained by 2D-NMR HMBC pulse sequence).

```
                                             Overall  InnerShell  OuterShell
Low resolution limit                           38.01     38.01      4.06
High resolution limit                           3.70      9.07      3.70

Rmerge  (within I+/I-)                         0.565     0.259     0.718
Rmerge  (all I+ and I-)                        0.586     0.262     0.746
Rmeas (within I+/I-)                           0.641     0.288     0.812
Rmeas (all I+ & I-)                            0.628     0.281     0.797
Rpim (within I+/I-)                            0.299     0.125     0.377
Rpim (all I+ & I-)                             0.220     0.096     0.275
Rmerge in top intensity bin                    0.229        -         -
Total number of observations                    9688       716      2198
Total number unique                             1250       104       268
Mean((I)/sd(I))                                  2.6       5.0       2.0
Mn(I) half-set correlation CC(1/2)             0.928     0.962     0.914
Completeness                                    97.7      99.6      92.0
Multiplicity                                     7.8       6.9       8.2
Mean(Chi^2)                                     0.95      0.81      0.81

Anomalous completeness                          97.9     100.0      93.0
Anomalous multiplicity                           4.5       5.0       4.6
DelAnom correlation between half-sets         -0.163    -0.447    -0.144
Mid-Slope of Anom Normal Probability           0.821        -         -

The anomalous signal appears to be weak so anomalous flag was left OFF

Estimates of resolution limits: overall
   from half-dataset correlation CC(1/2) >  0.30: limit =  3.70A
   from Mn(I/sd) >  1.50:                         limit =  3.70A
   from Mn(I/sd) >  2.00:                         limit =  3.70A

Estimates of resolution limits in reciprocal lattice directions:
  Along h axis
   from half-dataset correlation CC(1/2) >  0.30: limit =  3.70A
   from Mn(I/sd) >  1.50:                         limit =  3.70A
  Along k axis
   from half-dataset correlation CC(1/2) >  0.30: limit =  3.70A
   from Mn(I/sd) >  1.50:                         limit =  4.46A
  Along l axis
   from half-dataset correlation CC(1/2) >  0.30: limit =  3.70A
   from Mn(I/sd) >  1.50:                         limit =  3.70A

Anisotropic deltaB (i.e. range of principal components), A^2: 36.11

Average unit cell:    36.15   51.83   55.91   90.00   90.00   90.00
Space group: P 21 21 21
Average mosaicity:   0.66
```

Table S4: Crystallographic data collection statistics. Diffraction images were indexed and integrated with the XDS software. Reflection intensities were merged, and crystallographic data collection statistics calculated with Aimless (CCP4 suite).

Supplementary References

64 A. J. Nicoll et al., Pharmacological chaperone for the structured domain of human prion protein. Proc Natl Acad Sci U S A 107, 17610-17615 (2010).

65 J. Torrent et al., Pressure reveals unique conformational features in prion protein fibril diversity. Sci Rep. Feb 26;9(1):2802 (2019).
