# Supplementary material for: Pharmacological inactivation of the prion protein by targeting a folding intermediate
Source: Commun Biol. 2021 Jan 12;4:62. doi: 10.1038/s42003-020-01585-x (PMC7804251; doi:10.1038/s42003-020-01585-x)
Supplement: Supplementary file 3 — Description of Additional Supplementary Files [file 42003_2020_1585_MOESM3_ESM.pdf]

Description of Additional Supplementary Files

File Name: Supplementary Movie 1
Description: Full-atomistic reconstruction of PrP folding. Visualization of a representative least biased trajectory reconstructing the entire sequence of events leading the PrP polypeptide to reach the native state.
