# Supplementary material for: Pharmacological inactivation of the prion protein by targeting a folding intermediate
Source: Commun Biol. 2021 Jan 12;4:62. doi: 10.1038/s42003-020-01585-x (PMC7804251; doi:10.1038/s42003-020-01585-x)
Supplement: Supplementary file 5 — Reporting Summary [file 42003_2020_1585_MOESM5_ESM.pdf]

nature research

Corresponding author(s): Emiliano Biasini

Last updated by author(s): Dec 6, 2020

# Reporting Summary

Nature Research wishes to improve the reproducibility of the work that we publish. This form provides structure for consistency and transparency in reporting. For further information on Nature Research policies, see our Editorial Policies and the Editorial Policy Checklist.

## Statistics

For all statistical analyses, confirm that the following items are present in the figure legend, table legend, main text, or Methods section.

| n/a | Confirmed | |
|---|---|---|
| ☐ | ☒ | The exact sample size (*n*) for each experimental group/condition, given as a discrete number and unit of measurement |
| ☐ | ☒ | A statement on whether measurements were taken from distinct samples or whether the same sample was measured repeatedly |
| ☐ | ☒ | The statistical test(s) used AND whether they are one- or two-sided<br>*Only common tests should be described solely by name; describe more complex techniques in the Methods section.* |
| ☐ | ☒ | A description of all covariates tested |
| ☐ | ☒ | A description of any assumptions or corrections, such as tests of normality and adjustment for multiple comparisons |
| ☐ | ☒ | A full description of the statistical parameters including central tendency (e.g. means) or other basic estimates (e.g. regression coefficient) AND variation (e.g. standard deviation) or associated estimates of uncertainty (e.g. confidence intervals) |
| ☐ | ☒ | For null hypothesis testing, the test statistic (e.g. $F$, $t$, $r$) with confidence intervals, effect sizes, degrees of freedom and $P$ value noted<br>*Give P values as exact values whenever suitable.* |
| ☒ | ☐ | For Bayesian analysis, information on the choice of priors and Markov chain Monte Carlo settings |
| ☒ | ☐ | For hierarchical and complex designs, identification of the appropriate level for tests and full reporting of outcomes |
| ☒ | ☐ | Estimates of effect sizes (e.g. Cohen's *d*, Pearson's *r*), indicating how they were calculated |

*Our web collection on statistics for biologists contains articles on many of the points above.*

## Software and code

Policy information about availability of computer code

| | |
|---|---|
| Data collection | Molecular dynamics simulations were performed with Gromacs 4.6.5 (www.gromacs.org) with the plugin Plumed 2.0.2 (https://www.plumed.org) and with Gromacs 2018. |
| Data analysis | Data analysis was performed with the software R-Studio (version 1.1.414, https:/rstudio.com), Chimera (version 1.12, www.cgl.ucsf.edu) and Prism (version 7.0, GraphPad, www.graphpad.com). Additional analyses were performed using Python (version 2.7, www.python.org). |

For manuscripts utilizing custom algorithms or software that are central to the research but not yet described in published literature, software must be made available to editors and reviewers. We strongly encourage code deposition in a community repository (e.g. GitHub). See the Nature Research guidelines for submitting code & software for further information.

## Data

Policy information about availability of data

All manuscripts must include a data availability statement. This statement should provide the following information, where applicable:

- Accession codes, unique identifiers, or web links for publicly available datasets
- A list of figures that have associated raw data
- A description of any restrictions on data availability

The data that support the findings of this study are available withinin the manuscript or from the corresponding authors upon reasonable request.

# Field-specific reporting

Please select the one below that is the best fit for your research. If you are not sure, read the appropriate sections before making your selection.

☒ Life sciences ☐ Behavioural & social sciences ☐ Ecological, evolutionary & environmental sciences

For a reference copy of the document with all sections, see nature.com/documents/nr-reporting-summary-flat.pdf

# Life sciences study design

All studies must disclose on these points even when the disclosure is negative.

| | |
|---|---|
| Sample size | The sample size (n) of each experiment is provided in the corresponding figure captions in the main manuscript and supplementary information files. Sample sizes were chosen to support meaningful conclusions. |
| Data exclusions | Statistical analyses included all the data points obtained, with the exception of experiments in which negative and/or positive controls did not give the expected outcome, which were discarded. |
| Replication | All experiments were replicated at least three times, unless indicated differently |
| Randomization | Randomization of samples was not applicable to the reported experiments. |
| Blinding | All the data were collected and analyzed blindly by two different operators. |

# Reporting for specific materials, systems and methods

We require information from authors about some types of materials, experimental systems and methods used in many studies. Here, indicate whether each material, system or method listed is relevant to your study. If you are not sure if a list item applies to your research, read the appropriate section before selecting a response.

## Materials & experimental systems

| n/a | Involved in the study | |
|---|---|---|
| ☐ | ☒ | Antibodies |
| ☐ | ☒ | Eukaryotic cell lines |
| ☒ | ☐ | Palaeontology and archaeology |
| ☒ | ☐ | Animals and other organisms |
| ☒ | ☐ | Human research participants |
| ☒ | ☐ | Clinical data |
| ☒ | ☐ | Dual use research of concern |

## Methods

| n/a | Involved in the study | |
|---|---|---|
| ☒ | ☐ | ChIP-seq |
| ☒ | ☐ | Flow cytometry |
| ☒ | ☐ | MRI-based neuroimaging |

## Antibodies

| | |
|---|---|
| Antibodies used | Anti-PrP antibodies D18 (kindly provided by D. Burton, The Scripps Research Institute, La Jolla, CA) and 6D11 (Covance, catalog # SIG-399810); anti-NEGR-1 (kindly provided by Giovanni Piccoli, University of Trento, Italy); anti-Thy-1 (OX7, Abcam); anti-LC3 (ab51520, Abcam). |
| Validation | All reported antibodies have extensively been validated in previous pubblications, all appropriately referenced into the manuscript. |

## Eukaryotic cell lines

Policy information about cell lines

| | |
|---|---|
| Cell line source(s) | HEK293 and N2a cells were obtained from ATCC (ATCC CRL-1573 and CCL-131, respectively). We used a subclone (A23) of HEK293 stably expressing a mouse wild-type PrP or an EGFP-PrP construct, both already described and characterized previously (ref 57). L929 mouse fibroblasts and inducible RK13 cells were kindly provided by Ina Vorberg (DZNE, Bonn, Germany, ref. 58) and Didier Villette (INRA, Toulouse, France, ref. 59), respectively. Human cancer cell lines (H358, ZR-75, A549, H460, MCF7, H1299, SKBR3 and T47D), all belonging to the NCI collection of human cancer cell lines, were kindly provided by Valentina Bonetto (Mario Negri Institute, Milan, Italy). |
| Authentication | None of the cell lines were authenticated by the authors. |
| Mycoplasma contamination | All cell lines were tested negative for mycoplasma contamination by the Cell and Tissue Facility at CIBIO, University of Trento, Italy. |

| | |
|---|---|
| Commonly misidentified lines<br>(See ICLAC register) | Not applicable. |
